# Rapid prediction of conformationally-dependent DFT-level descriptors using graph neural networks for carboxylic acids and alkyl amines†

**DOI:** 10.1039/d4dd00284a

**Published:** 2024-11-28

**Authors:** Brittany C. Haas, Melissa A. Hardy, Shree Sowndarya S. V., Keir Adams, Connor W. Coley, Robert S. Paton, Matthew S. Sigman

**Affiliations:** a Department of Chemistry, University of Utah Salt Lake City Utah 84112 USA matt.sigman@utah.edu; b Department of Chemistry, Colorado State University Fort Collins Colorado 80523 USA robert.paton@colostate.edu; c Department of Chemical Engineering, Massachusetts Institute of Technology Cambridge Massachusetts 02139 USA ccoley@mit.edu; d Department of Electrical Engineering and Computer Science, Massachusetts Institute of Technology Cambridge Massachusetts 02139 USA

## Abstract

Data-driven reaction discovery and development is a growing field that relies on the use of molecular descriptors to capture key information about substrates, ligands, and targets. Broad adaptation of this strategy is hindered by the associated computational cost of descriptor calculation, especially when considering conformational flexibility. Descriptor libraries can be precomputed agnostic of application to reduce the computational burden of data-driven reaction development. However, as one often applies these models to evaluate novel hypothetical structures, it would be ideal to predict the descriptors of compounds on-the-fly. Herein, we report DFT-level descriptor libraries for conformational ensembles of 8528 carboxylic acids and 8172 alkyl amines towards this goal. Employing 2D and 3D graph neural network architectures trained on these libraries culminated in the development of predictive models for molecule-level descriptors, as well as the bond- and atom-level descriptors for the conserved reactive site (carboxylic acid or amine). The predictions were confirmed to be robust for an external validation set of medicinally-relevant carboxylic acids and alkyl amines. Additionally, a retrospective study correlating the rate of amide coupling reactions demonstrated the suitability of the predicted DFT-level descriptors for downstream applications. Ultimately, these models enable high-fidelity predictions for a vast number of potential substrates, greatly increasing accessibility to the field of data-driven reaction development.

## Abstract 2

DFT-level descriptor libraries were constructed to train 2D and 3D graph neural networks for on the-fly the prediction of carboxylic acid and alkyl amine descriptors suitable for statistical modeling of medicinally relevant molecules.

## Graphical abstract

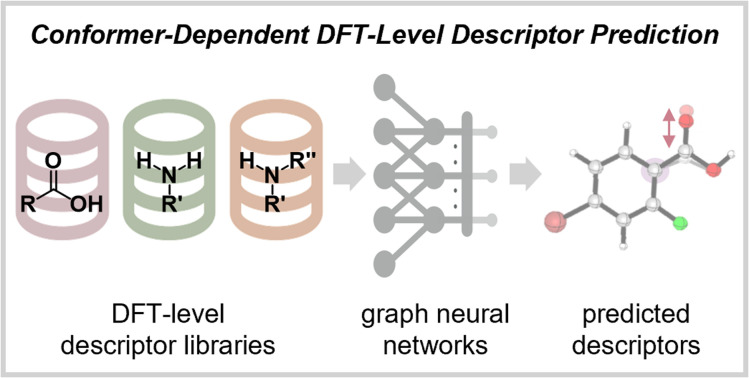

## Introduction

Data science is emerging as a means to probe structure–activity relationships, design and analyze chemical space, and optimize chemical reactions, ultimately impacting a range of applications in the chemical enterprise.^2^ However, the computational infrastructure required to do so can be prohibitive to laboratories with experimental data but limited experience with and/or access to high performance computing (HPC) resources. To combat this issue, density functional theory (DFT)-level molecular descriptor libraries for commonly used substrates,^3,4^ ligands,^5–7^ and drug-like molecules^8^ have been constructed and disseminated to reduce redundancy of expensive calculations in the field. Descriptor libraries can be built agnostic to a particular reaction, and thus can be used in a range of applications; libraries of this type have been demonstrated to guide the selection of diverse reaction substrates,^4,9,10^ predict the outcome (*e.g.*, selectivity,^11–13^ rate,^14^ or yield^15^) of chemical reactions, and elucidate key mechanistic features.^11,12,16^ In particular, we have found success applying atom- or bond-level descriptors focused on conserved moieties in each reaction component of a dataset, which are hypothesized to lend specific insight into the reactive site in order to maximize interpretability and mechanistic understanding.

While published descriptor libraries may attempt to incorporate common substrates or ligands, it is not practical (or possible) to precompute DFT-level descriptors for every compound a user may wish to featurize. For successful dataset design or predictive modeling, the defined descriptor set needs to be calculated for each new compound of interest. Replicating a full descriptor calculation workflow to add even a single compound to the library can require tedious coordination of numerous software packages, license agreements, computing clusters, *etc.* Automated workflows to perform these tasks^17–20^ can mitigate several of these challenges but do not circumvent the computational cost. Thus, it would enable downstream applications if an existing library could be exploited to predict relevant descriptors for new compounds within seconds without requiring additional calculations.

Across the chemical sciences, machine learning (ML) models have demonstrated the ability to serve as surrogates for electronic structure calculations and other simulation techniques connecting molecular structures to computed properties. Previously, prediction of DFT-level descriptors has been accomplished for single properties on a broad range of molecules.^21–30^ Additionally, ML property prediction has been used to expand a DFT-level descriptor library of monophosphines 200-fold by combinatorializing substructures present in the existing library.^6^

**Fig. 1:**
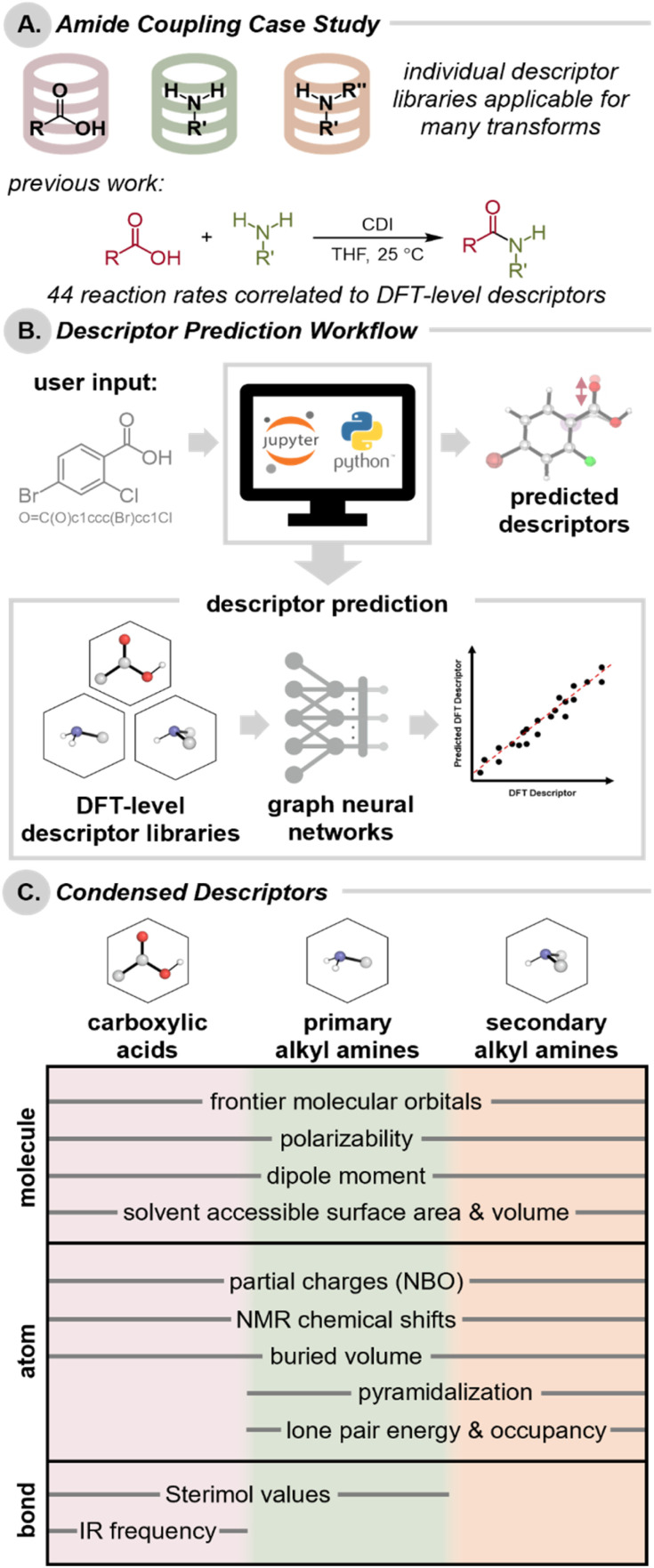
(A) Carboxylic acid, primary amine, and secondary amine descriptor libraries as applied to amide coupling reactions. (B) Workflow for predicting descriptors from SMILES strings. (C) DFT-level descriptors predicted for each library, denoted as molecule-, bond, or atom-level properties.

Herein, we describe a case study investigating the prediction of a set of conformationally-informed descriptors collected for a single conserved reactive moiety—either a carboxylic acid or a primary/secondary alkyl amine (Fig. 1A). Our selection of carboxylic acids and amines was motivated by the ubiquity of amide couplings in medicinal chemistry,^31,32^ as well as our recent efforts to correlate the reaction rates of amide couplings with DFT-level molecular descriptors of carboxylic acid derivatives and primary alkyl amines.^14^ We envisioned a method wherein a user would be able to simply supply a SMILES string (or draw a chemical structure) to obtain high-fidelity predictions of DFT-level descriptors for use in downstream applications without the need for HPC resources (Fig. 1B). To obtain accurate predictions, we needed to address the challenge of predicting diverse descriptors (*i.e.*, steric, electronic, and stereoelectronic properties at the molecule-, bond-, and atom-level) that account for the dynamic range of properties stemming from the conformational flexibility of compounds in these classes (Fig. 1C).^33–36^ We applied graph neural networks (GNNs) trained on expansive libraries to predict these diverse DFT-level descriptors. We demonstrated these predicted descriptors are appropriate for data-driven modeling of medicinally-relevant carboxylic acid and alkyl amines. Moreover, Hammett parameters for aryl carboxylic acids are a cornerstone of physical organic chemistry;^37–39^ therefore, we envision an extensive library of carboxylic acid descriptors should have widespread applications as surrogate descriptors. The rapid prediction of descriptors also greatly expands the direct applicability of the carboxylic acid and alkyl amine descriptor libraries, reducing the barrier of entry to dataset design and predictive modeling.

## Results and discussion

### Descriptor library building

**Fig. 2:**
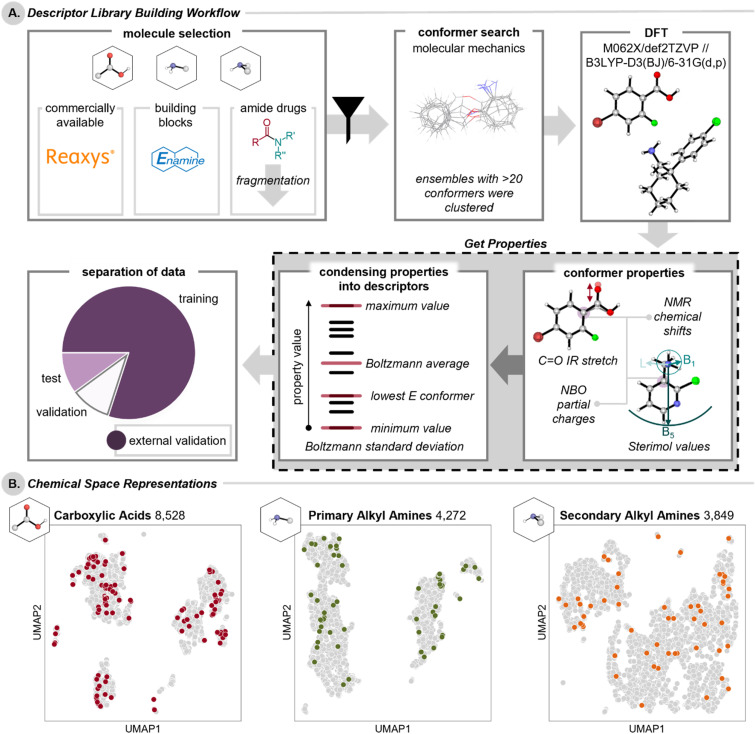
(A) Workflow for the construction of carboxylic acid, primary alkyl amine, and secondary alkyl amine descriptor libraries. (B) UMAP chemical space representations of the descriptor libraries; compounds in the external validation set are highlighted. Projections were calculated for each subpanel separately.

In order to obtain representative libraries of carboxylic acids and amines, the Reaxys database^40^ was queried to identify commercially available carboxylic acids, primary alkyl amines, and secondary alkyl amines that would be applicable to amide coupling reactions (Fig. 2A).§§Commercial availability was identified by the Reaxys filter but not verified or limited to particular suppliers. Examples of excluded functional groups include alcohols, thiols, and salts. See ESI Section 1.1† for a full list of exclusions for each class. To ensure broad representation of molecules relevant to medicinal chemistry for downstream library applications, external validation sets of acids and amines were also compiled from Enamine's building block sets^41^ and from acid and amine fragments of existing amide-containing drugs mined from the Broad Institutes Drug Repurposing Hub.^42^ The full lists of compounds are available on Figshare.^1^

Previous studies have revealed the utility of molecular descriptors that encompass the dynamic range of conformers that a molecule can adopt within a given energetic window.^6^ To this end, we conducted automated conformational searching and clustering with Schrodinger's Maestro^43^ to access representative conformational ensembles for each compound, which were then further optimized using the Gaussian 16 software at the M06-2X/def2-TZVP-SDD(I, Sn, Se)//B3LYP-D3(BJ)/6-31G(d,p)-LANL2DZ(I, Sn, Se) level of theory.^44–50^ Natural bond orbitals (NBO) and spectroscopic parameters were further evaluated at the DFT level of theory on these optimized geometries. Gas phase calculations provide molecular descriptors that are compatible with a variety of solvents and have generally proven sufficient for statistical modeling of chemical reactions.^14^ To process these calculations, a *Get Properties* jupyter notebook was developed to enable the collection of descriptors at the molecule-level, in addition to atom- and bond-level properties for a conserved moiety of interest.^51^ Specifically, for each of these three libraries, we collected global properties (*e.g.*, frontier molecular orbital energies of the HOMO and LUMO, polarizability, dipole moment, solvent accessible surface area, and solvent accessible volume) of the molecule, and tabulated the atom-level properties (*e.g.*, NBO natural population analysis partial charge, NMR chemical shift, and buried volume) of conserved atoms (Fig. 1C). In the case of the amine libraries, additional atom-level properties relevant to the nitrogen of the amine (*i.e.*, pyramidalization, lone pair energy, and lone pair occupancy) were also collected. Bond-level Sterimol values were calculated for both the acid and the primary amine moieties to give insight into the steric environment of their substituents.¶¶For secondary amines, no atom or bond properties were collected using C^2^ or C^3^ due to the inability to differentiate between symmetrical groups; for primary amines, atom properties collected for H^3^ and H^4^ were combined to give the average property value for the two atoms for the same reason. Additionally, for the acid library, the IR harmonic stretching frequency of the carbonyl was compiled.

For a given conformational ensemble, the minimum property value, the maximum property value, the property value from the lowest energy conformer, and Boltzmann-weighted average and standard deviation were calculated. These condensed descriptors encompass both the extreme conformers a molecule can adopt (within an allowable energetic window of 5 kcal mol^−1^) as well as accessible, aggregate conformations. It can be important to represent conformational flexibility, as the active conformer in a given transformation cannot be determined *a priori*; thus, it is difficult to hypothesize which condensed descriptor will be deemed important/insightful. Descriptors of this type have been used to classify ligation states^34^ and have since gained traction in uncovering reactivity trends.^52,53^ Of note, the *Get Properties* notebook, available on GitHub (https://github.com/SigmanGroup/Get_Properties/), is automated and adaptable to facilitate descriptor collection for any conserved moiety with a SMARTS string input.^54^ Similar automated workflows (utilizing various software packages) from the Doyle^17^ and the Paton^18^ groups have also been developed to generate conformers and collect DFT-derived descriptors, often extracting all possible descriptors rather than focusing on a single moiety.

These efforts resulted in three libraries: (1) 8528 carboxylic acids encompassing 71 324 unique conformers to provide 275 ensemble-based descriptors, (2) 41 452 conformers for 4272 primary alkyl amines provided 170 descriptors, and (3) 39 207 conformers for 3849 secondary alkyl amines provided 145 descriptors. All conformer properties and ensemble descriptors are provided on Figshare.^1^ To evaluate models for these DFT-level descriptors, each library was randomly divided into a training, a validation, and a test set, with the Enamine- and Drug Repurposing Hub-derived^41,42^ external validation set held back for final model evaluation (acids: 7301/480/476/149,||||From the full acid library, several acids were removed for execution of 2D GNNs due to openbabel parsing errors. primary amines: 3209/500/500/63, secondary amines 2798/500/500/51, combined amines 6007/1000/1000/114). The dimensionality reduction technique uniform manifold approximation and projection (UMAP)^55^ was used to provide a 2D chemical space representation of each of these libraries (Fig. 2B).**Given the similarities between the descriptors collected for primary and secondary alkyl amines, it is feasible to build a combined library with the conserved descriptors and map its chemical space. In practice, we found that there was almost no overlap between primary and secondary amines, see ESI Section 3.4† for full details. The external validation sets of medicinally relevant acids and amines were mapped onto their respective chemical spaces, showing that such molecules are similar to those in our libraries.

These libraries would be poised for application in numerous unique reaction development campaigns, including training set design, as well as statistical modeling and subsequent virtual screening with mechanistically interpretable descriptors. However, given the expansive nature of substrate variations, it is probable that a user will be interested in a compound not included in the library. While DFT calculations of additional acids and amines could be performed, this would not be feasible to do on-the-fly for large virtual screening campaigns, thus motivating our interest in predicting DFT-level descriptors.

### 2D graph neural networks for descriptor prediction

GNNs are well-suited to supervised learning problems involving graph-structured data and align well with the task at hand of predicting features from a molecular structure. Early modeling efforts showed multi-task modeling^56^ and fingerprint-based gradient boost regressor (GBR) models (ESI Section 4.4†) to be unsuccessful. However, previous studies on molecular descriptor prediction had found success employing 2D/3D graph inputs (*i.e.*, without conformational ensemble information embedded) for properties such as chemical shift^21^ and bond dissociation energy.^22,23^ As such, we first aimed to evaluate a message-passing GNN employing 2D-input representations for our application to predict condensed descriptors encompassing the dynamic range of accessible conformers and their electronic, steric, and stereoelectronic properties.

**Fig. 3:**
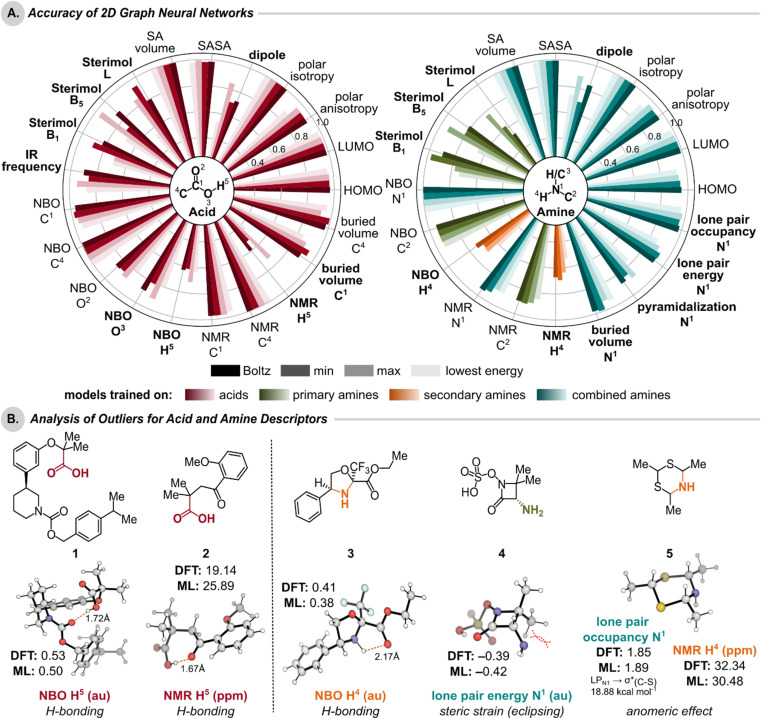
(A) Model performance determined by the test set (*R*^2^) for the prediction of condensed descriptors (Boltzmann-averaged properties, minimum and maximum property values, lowest energy conformer's property value) of carboxylic acids and alkyl amines. 3D GNNs were trained for descriptors with bolded labels. Bar colors indicate the dataset that the descriptor model was trained on. The performance of all models trained using the combined amine sets was also evaluated for the combined amine set. Evaluation of the individual amine subsets is presented on Figshare.^1^ (B) Acid and amine test set compounds whose 2D GNN predicted Boltzmann-averaged descriptors most significantly deviate from the DFT-derived descriptor value were identified. The lowest energy conformers, which contribute most to the Boltzmann-averaged descriptor, are depicted.

The Boltzmann-weighted average (Boltz), minimum (min), maximum (max), and lowest energy conformer (low E) descriptors are normally distributed for most properties (ESI Section 2.2†) and describe a diverse set of molecules (ESI Section 2.3† for atom diversity in libraries). The atoms of interest correspond to the conserved atoms in the (R)_3_C^4^–C^1^O^2^O^3^H^5^ acid functional group, (R)C^2^–N^1^H^3^H^4^ in the primary amines, and (R)_2_N^1^H^4^ in the secondary amines (Fig. 3A).

Each molecule was encoded as a 2D molecular graph where the nodes/edges correspond to atoms/bonds, respectively. Using the open-source cheminformatics package RDKit,^57^ information on the type of atom, valence electrons, chirality, formal charge, aromaticity, ring information, degree, and total number of hydrogens bonded to each atom was one-hot encoded into each node of the graph. Similar information for the bond including bond-type, conjugation, ring information, and stereochemistry was one-hot encoded to the edges of the molecular graph (all features are listed in the ESI Section 4.2†). These molecular graph inputs were used for training a neural network where the node and edge representations were updated at each layer based on neighboring nodes and edges, allowing for information flow within the molecular graph. This information flow (*i.e.*, message-passing) was performed using a graph isomorphism network with edge features (GINE) convolutions,^58^ and the final representations were utilized to make predictions of atom-, bond-, and molecule-level descriptors. (More information on the architecture can be found in ESI Section 4.1†).

We trained one GNN for each molecule-, bond-, and atom-level descriptor individually, and evaluated model accuracy using the withheld test sets for both the acids and amines.^51^ For the primary and secondary amines, models for the conserved descriptors for both functional groups (*e.g.*, HOMO, N^1^ NMR, N^1^ NBO, *etc.*) were trained using the combined dataset, while models for descriptors that were only collected for primary amines (*i.e.*, Sterimol values, C^4^ NMR, C^4^ NBO) or secondary amines (*i.e.*, H^4^ NMR and H^4^ NBO) were trained solely on their respective libraries.††††For primary amines, no atom or bond properties were modeled using H^3^ and H^4^, as these properties were reported as the average of the two atoms in the library.

For each molecule-level descriptor, the models achieved high accuracy (*R*^2^ > 0.95) across the condensed descriptor set for both acids and amines, with the exception of the dipole moment.‡‡‡‡Lower accuracies for dipole moment can be attributed to dependence of conformers, the directional aspect of dipole which has failed to be captured by the GNNs. For the atom-level descriptors of acids, the models obtained higher accuracy (*R*^2^ > 0.94) for NBO partial charges on C^1^ and C^4^ compared to those on O^2^, O^3^, and H^5^ (0.61 < *R*^2^ < 0.91). Upon analyzing the outliers for NBO partial charges, we noted that hydrogen-bonding had a significant impact on the charges of O^2^, O^3^, and H^5^. To investigate prediction failures, the lowest energy conformers were considered, as they have the greatest influence on the Boltzmann-averaged descriptor. For example, the acid molecule with the maximum deviation showed that our model underpredicts the charge on H^5^ and may not account for the increase in positive charge on the H^5^ atom attributed to H-bonding (1, Fig. 3B). Similar trends were observed for NMR chemical shifts, where we noted enhanced performance for C^1^ and C^4^ compared to H^5^ (*R*^2^ > 0.96 *vs. R*^2^ < 0.73), which can again be ascribed to the presence of an intramolecular H-bond (2).§§§§The error reported for H^5^ NMR has a predicted value much larger than the DFT-calculated shift. Additional analysis of nearest neighbors of the H^5^ NMR chemical shift outlier is reported in ESI Section 4.3.† Models for amine atom-level descriptors such as NBO partial charges on N^1^ and C^2^ achieved high accuracy (*R*^2^ > 0.94) but exhibited poorer performance for H^4^ (*R*^2^ < 0.74). Similar to the carboxylic acids, H-bonding impacts the charge on the H^4^ atom of amine 3. Additionally, model performance was compromised for the lone pair energy of the N^1^ atom. Upon analyzing the most extreme outlier (4), destabilizing interactions due to eclipsing lone pair and methyl substituents increase the lone pair energy. With respect to NMR chemical shifts, while adequate performance is obtained for N^1^ and C^2^ (*R*^2^ > 0.85), H^4^ shifts had significantly lower *R*^2^ values (<0.69). The most extreme outlier was due to an anomeric effect caused by the back donation of LP_N1_ to 
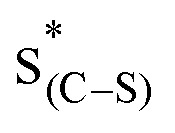
 (interaction energy of 18.88 kcal mol^−1^ obtained from second-order perturbation analysis of the Fock matrix in the NBO basis), which deshields the proton (H^4^) (5). The same compound was also an outlier in the prediction of N^1^ lone pair occupancy, wherein the reduction in occupancy is found due to back-donation. These examples highlight the limitations of using simple 2D representations that may fail to capture critical stereoelectronic effects.

However, for the acid and amine bond-level descriptors Sterimol L, B_1_, and B_5_ values, the models obtain varied accuracies. This can be partially explained by the pronounced directional and conformational dependence of these descriptors. Because the GNNs employ 2D molecular representations, they may be less adept at capturing conformational- and directionality-based information to which Sterimol descriptors are acutely sensitive.¶¶¶¶Detailed analysis of which descriptors are most conformationally dependent is reported in ESI Section 2.4.† Hence, significant differences in Sterimol L, B_1_, and B_5_ values were observed, even within Boltzmann-averaged, minimum, maximum, and lowest energy conformer properties for a single descriptor.

Mean absolute error (MAE) and *R*^2^ metrics for each acid and amine descriptor predicted for the test set can be found on Figshare.^1^ Overall, while 2D-based modeling has shown high predictive accuracies for molecular descriptors of acids and amines, we hypothesized that more information-rich molecular representations capturing aspects of 3D geometry would improve accuracies for atom- and bond-level descriptors particularly sensitive to stereoelectronic effects or 3D molecular structure generally.

### Incorporating 3D-geometries for descriptor prediction

In contrast to 2D GNNs, which solely encode the information stored in a graph representation of a molecule and pass messages between covalently bonded atoms, 3D GNNs generally encode point clouds representing the coordinates of a specific 3D conformation of the molecule. Under this paradigm, edges in the 3D graph no longer solely correspond to covalent bonds, but additionally include non-covalent edges between any two atoms separated by a distance within a defined cutoff. By explicitly encoding the local geometry of these 3D graphs (*e.g.*, in terms of distances, angles, and dihedrals), 3D GNNs have the capacity to directly capture 3D geometric features present in the conformers provided as input to the model. We hypothesized that this enhanced geometric expressivity would improve the surrogate descriptor prediction models, particularly for geometry-sensitive descriptors such as Sterimol values and NBO partial charges.

The requirement of input conformers when using 3D GNNs introduces additional questions with regards to how those conformers should be generated. Applying DFT-level conformers, such as those used to compute the target acid/amine descriptors, would offset the desired cost savings of using a ML surrogate, so we instead employed cheap-to-acquire conformers that are separately embedded with the ETKDG algorithm^59^ and optimized with MMFF94,^60^ all *via* RDKit.^57^ For the relatively low molecular weight acids and amines studied here, this conformer generation was fast and accessible for thousands of molecules with limited computational resources (*ca.* 2.5 CPU seconds per molecule). Our use of MMFF94 conformers assumes that these structures provide an adequate representation of the ground-truth DFT-optimized conformer ensemble, which we later show to be empirically defensible, albeit imperfect. For instance, the cheap surrogate conformers may not exhibit the same stereoelectronic structural features present in the DFT-optimized ensembles, which would reduce the capability of the 3D GNNs to capture such effects. While this risk could potentially be mitigated by exploring the use of higher-cost optimization strategies, here our priority was providing rapid and facile access to predictions of DFT-level descriptors. In cases where there were multiple MMFF94 conformers for a given molecule, we used up to 20 conformers as a form of data augmentation during model training, mapping each individual MMFF94 structure to the same descriptor of the DFT-optimized ensemble. This data augmentation strategy has been shown to modestly improve the modeling of conformer ensembles with little additional computation cost apart from conformer generation.^61^ Further details on our conformer generation workflow can be found in the ESI Section 5.2.†

As the base 3D GNN architecture, we used DimeNet++,^62^ which consistently performs on-par with or better than significantly more expensive equivariant 3D GNNs, especially for property prediction in relatively low-data tasks.^61^ To make use of atom features, we replaced DimeNet++’s default atom featurizer with the same one-hot encoded features employed in our 2D GNNs, but bonds were not featurized. Furthermore, when applying each 3D GNN, we averaged the model's predictions over <20 MMFF94 conformers to further reduce any noise relating to the choice of input conformer at test-time. Otherwise, the output of the 3D GNNs and the associated training protocols were analogous to those of the 2D GNNs.

**Fig. 4:**
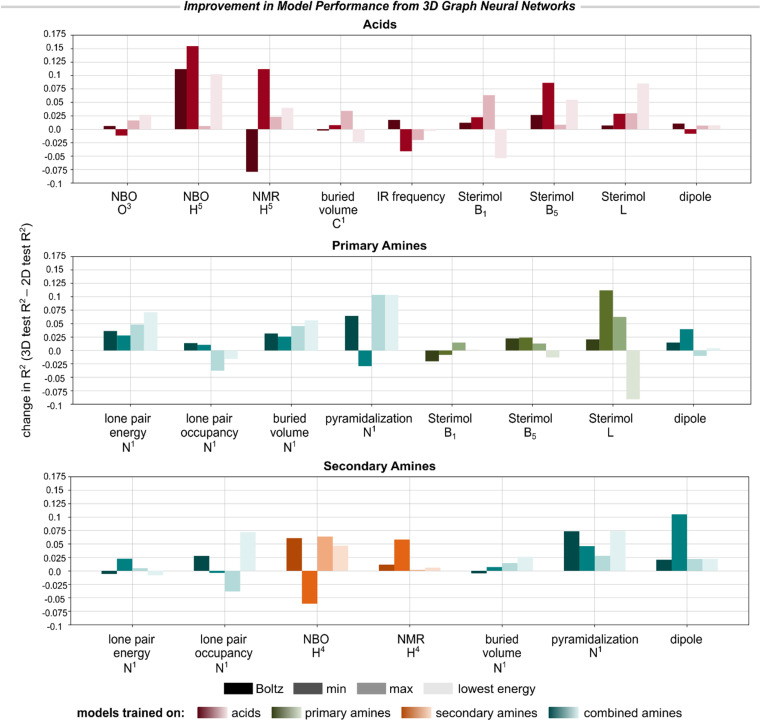
Improvement in model performance on select molecule-, bond-, and atom-level descriptors when using the 3D GNN *versus* the 2D GNN, reported as the change in *R*^2^ for the test set. See ESI Section 5.4† for reported improvements in MAE for each property. Due to the non-overlapping chemical spaces of the primary and secondary amines, we report model performance on each subset individually.

Fig. 4 reports the improvement in each 3D model's performance (*R*^2^) compared to the corresponding 2D GNN when evaluated on the same test set.||||||||A number of compounds could not be parsed with openbabel or embedded with RDKit, and hence the 3D GNNs were trained on marginally smaller datasets than the 2D GNNs (acids: 7290/478/476/149, primary amines: 3168/495/494/63, secondary amines 2425/500/498/51, combined amines 5593/995/992/114).Fig. 4 compares the models when evaluated on the exact same test data for which conformers could be embedded. Due to the greater cost associated with training the 3D models, we focused on modeling only a subset of descriptors for which the 2D GNNs had relatively poor accuracy, especially properties exhibiting sensitivity to stereoelectronic or geometric effects.^51^ In general, we found that the 3D GNN—even when using MMFF94-level conformers—led to slight or substantial improvement in model accuracy for the majority of the modeled descriptors. The most significant improvements were observed for hydrogen atom NBO and NMR descriptors, Sterimol values, and amine pyramidalization. Only marginal improvement or diminished performance was observed for buried volumes, dipole moments, lone pair descriptors for the amines, and IR frequencies of the acids.

Moreover, with the exception of IR frequencies, the 3D GNNs always achieved lower MAE, even if the *R*^2^ did not improve (or was worsened), suggesting that encoding 3D molecular geometries improves the accuracy of descriptor prediction on average but may lead to greater sensitivity to outliers. Interestingly, we found that two of the six outliers (*i.e.*, amine N^1^ lone pair energy and amine H^4^ NMR) identified for the 2D GNN in Fig. 3B were predicted with substantially higher accuracy by the corresponding 3D GNN, but the remaining four cases saw little to no improvement by 3D modeling (see ESI Section 5.3†). It is worth noting that DFT-derived descriptors have some inherent noise associated with them due to various sources of stochasticity during structure generation, geometry optimization, and conformer clustering. For geometry-sensitive properties like Sterimol values, error from the descriptors will propagate into the model, negatively impacting predictive accuracy. This noise may especially impact the descriptors of the lowest-energy conformers, which generally saw significantly reduced predictive accuracy for both the 2D and 3D models. The imperfect ability of the 3D GNNs to account for instances of stereoelectronic effects may also be due to the use of surrogate MMFF94 conformers as model inputs, or simply due to the rarity of these interactions in the training data.

Additionally, we confirmed that these models could give predictions exponentially faster (*ca.* 10^5^ for 2D models and *ca.* 10^4^ for 3D models) than collecting descriptors through the DFT workflow. As an illustrative example, for the library of 8528 carboxylic acids, DFT calculations to build the library (not including conformer generation or property extraction) required over 1 000 000 CPU hours to complete. By contrast, predicting all descriptors for entire libraries of compounds is possible on a modern personal computer in approximately a single day; the library of carboxylic acid descriptors was predicted for all 8528 compounds from SMILES strings in 2 hours using the 2D models or in 13 hours using the 3D models with a single CPU.

### Evaluation of the external validation set

**Fig. 5:**
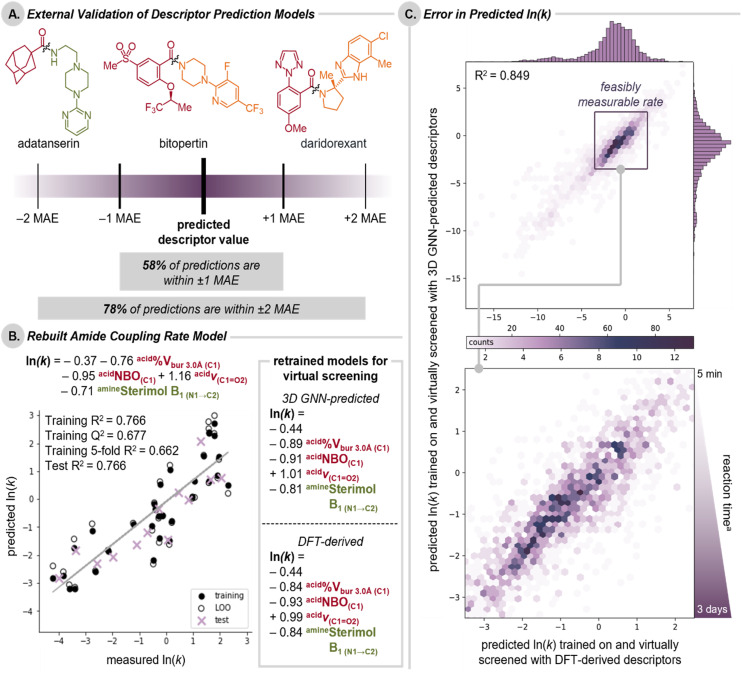
(A) Representative examples of the drug molecules within the external validation set and the percentage of total descriptor predictions within 1 or 2 test set MAE. (B) Rebuilt multivariate linear regression model for the rate of amide coupling reactions with CDI using the dataset published in *Proc. Natl. Acad. Sci. U.S.A.* 2022, 119, e2118451119. Analogous descriptors predicted from 3D GNNs published herein were used to train the model employing a 70 : 30 y-equidistant train : test split. Model equations used for virtual screening were refit to the unsplit dataset using either 3D GNN predicted descriptors or DFT-derived descriptors (right). (C) Hexbin plot of the predicted ln(*k*) of 2362 test and external validation set acid-primary amine couplings virtually screened using DFT-derived descriptors *vs.* 3D GNN predicted descriptors. The shading of the hexagons indicates the number of couplings in that region. The zoomed-in region represents couplings with rates that are experimentally feasible to measure. ^a^Reaction time to 97% conversion determined for 0.5 M reactions at 25 °C.

To further validate the descriptor prediction models for applications in medicinal chemistry, we specifically evaluated the external validation acids and amine sets. Example drug molecules adatanserin, bitopertin, and daridorexant, which have all been synthesized *via* amide coupling, highlight the complexity of compounds included in the external validation set (Fig. 5A).^63–65^ In general, descriptor models exhibited notably poorer performance on these complex fragments compared to our test set (Fig. 3 and 4) as measured by *R*^2^, however, we found that the MAEs of the test set and external validation set were typically comparable for each descriptor.^1^ We hypothesized the poor *R*^2^ values are due in part to the smaller dataset size that results in models that are more sensitive to outliers. Additionally, the relatively narrow range of descriptor values for the external validation set as compared to the rest of the library leads to a flat response surface for many descriptors.^66^ Overall, we found the majority of descriptors predicted for molecules in the external validation set were within one MAE (defined as the test MAE from 2D models or 3D models when available). This confirmed that the models suitably predict descriptors for drug-like molecules to facilitate their application in medicinal chemistry pursuits.

### Impact of descriptor error on downstream applications

In order to evaluate the error associated with predicted descriptors on downstream applications, we retrospectively analyzed a published statistical model for the rate of amide coupling reactions with CDI, which relies on DFT-level acid and primary amine descriptors.^14^ Of note, this example only includes substrate descriptors, as the model was trained on data collected under fixed reaction conditions; however, if expanded, models could be constructed to include descriptors for substrates, reagents, and other reaction conditions (*e.g.*, solvent, temperature, *etc.*). Furthermore, this case study should elucidate the impact of using predicted descriptors prospective studies where DFT-level descriptors are not available for every compound. We opted to use exclusively predicted descriptors rather than a mix of DFT-derived and predicted descriptors, to allow the model, in principle, to learn to cope with systematic errors in the predicted descriptors.****In practice, we found there was no particular advantage to using 3D-trained models over DFT-trained models when predicting amide coupling rates with 3D descriptors (ESI Section 6.3†).

Fig. 5B shows the previously published model rebuilt using the analogous 3D GNN predicted descriptors from the models published herein.††††††Analogous acid descriptors were used in place of the carboxylate and acyl imidazole model parameters. The C^1^ NBO Boltz 3D model was developed for this evaluation of error (not presented in Fig. 4). Of note, while models for condensed IR descriptors generally were not improved by implementing the 3D model architecture, the prediction of the Boltzmann-averaged IR descriptor was improved (as measured by both *R*^2^ and MAE). The model remains statistically similar and directly comparable to that in the original report. To further validate this approach, a virtual screening campaign was used to directly compare the use of models using DFT-derived or 3D GNN predicted descriptors. Comparing the two model equations trained on the entire dataset, similarities in the sign and magnitude of coefficients in the equations fit with DFT-derived and 3D GNN predicted descriptors highlight that both models lead to the same mechanistic conclusions regardless of the descriptor acquisition method used. For virtual screening, a total of 2362 amide couplings were generated by pairing acids and primary amines from the test and external sets.‡‡‡‡‡‡The full library is outside the scope of this model, but this simply serves as an exercise to demonstrate the acceptable error from predicted descriptors and how it propagates in their applications. Comparisons of rate predictions using exclusively DFT-derived or exclusively 3D GNN predicted descriptors revealed good overall agreement (*R*^2^ = 0.849, MAE = 0.719, Fig. 5C). At the extrema, error in predictions increases, but rate constants are either too fast to measure or slow to the point of no observable reaction in a reasonable timespan, making the predicted outcome functionally the same as no reaction or instantaneous, respectively. However, rate predictions within a realistic, experimentally measurable range of *k* (−3.5 < ln(*k*) < 2.5, 3 days to 5 min) exhibit less error (MAE = 0.573). Couplings with acetic acid were the only notably poor predictions and were excluded from the analysis.§§§§§§We expect this is due to the lack of small alkyl acid representation in the training set for descriptor prediction models (see ESI Section 6.5† for further discussion). Ultimately, we conclude that descriptors predicted using 3D GNNs are suitable for statistical modeling applications and valuable for conducting expansive virtual screens.

## Conclusion and outlook

In summary, we have developed fast, reliable models to predict DFT-level descriptors for carboxylic acids, primary alkyl amines, and secondary alkyl amines. We obtained high-accuracy predictions not only for descriptors containing aggregate information for the conformational ensemble (Boltzmann-averaged properties) but also for descriptors that encompass the extreme flexibility of these compounds (minimum and maximum property values). These models relied on the construction of large libraries of DFT-derived descriptors that are useful in and of themselves. To accomplish this, we have developed a user-friendly, automated script for collecting moiety-specific descriptors that have been key to mechanistically insightful modeling efforts.^11,12,54^

This case study further demonstrates the efficacy of applying 2D and 3D GNNs to predict DFT-level steric, electronic, and stereoelectronic descriptors of conformer ensembles from accessible molecular representations, such as the 2D molecular graphs or conformers derived from inexpensive molecular mechanics. While 2D models performed well for global molecule-level descriptors of the conformer ensembles, poorer performance was noted for local atom- and bond-level descriptors particularly sensitive to 3D geometry, directionality, or intramolecular non-covalent interactions. These difficult-to-predict descriptors include dipole moments, hydrogen NMR and NBO (for acids and amines), Sterimol values, and nitrogen pyramidalization and lone pair properties (for amines). To overcome these challenges, we developed 3D models that encode the geometries of readily accessible MMFF94-level conformers, striking a balance between geometric expressivity and the cost of conformer generation. The 3D models improved predictive accuracy for most descriptors, with notable gains in performance for hydrogen atom NMR and NBO descriptors, Sterimol values, and amine pyramidalization. Given the greater cost of the 3D models and conformer generation, we recommend the use of 2D GNNs for most molecule-level descriptors, atom-level descriptors without acute sensitivity to geometric characteristics, and other descriptors for which the 3D models worsen predictive accuracy, such as IR frequencies of acids. In contrast, using 3D GNNs can substantially enhance prediction performance for local atom- and bond-level descriptors sensitive to molecular geometries and/or non-covalent interactions, even when encoding computationally inexpensive conformers. In the future, additional modeling could focus on probing the limitations of predicting DFT-level descriptors from cheaper 3D representations, enhancing the ability of 2D and 3D models to capture intramolecular non-covalent through-space interactions, and generally improving the ability of GNNs to model the dynamic range of properties that result from conformer ensembles.

Each library is publicly hosted (https://descriptor-libraries.molssi.org/) for users to interactively investigate chemical space maps, identify nearest neighbors^67^ to substrates of interest, and design substrate scopes. These libraries could be used to develop “ideal” acid or amine substrates for evaluation, but for every unique application, it may be advantageous to further curate the space, either by identifying a subset of compounds relevant to the reaction of interest (*e.g.*, aryl/heteroaryl carboxylic acids) or by selecting a specific subset of descriptors based on a mechanistic hypothesis. Descriptors from these libraries can be downloaded to facilitate chemical space curation or to probe structure–activity relationships by building predictive models with these commonly employed substrate classes. Furthermore, once a predictive model has been identified and validated, expansive libraries of this type are poised to expedite virtual screening. For example, the libraries reported herein expand our previous study modeling the amide coupling reaction from 600 combinations of acids and amines calculated for modeling to enable virtual screening of over 32 million acid and amine combinations. Virtual screening is further expanded by these GNN models, which allow for the rapid prediction of condensed descriptors from a simple SMILES input for any user-designed compound.

Beyond applications relating directly to carboxylic acids and amines, it is worth noting that many of these carboxylic acid descriptors are expected to be directly correlated to descriptors for functional groups made from similar feedstocks (*e.g.*, aldehydes, esters, boronic acids, and sulfonic acids) that are commonly used in synthetic campaigns. Given that it has been demonstrated that DFT-calculated partial atomic charges and NMR chemical shifts,^39^ like those collected and predicted in this work, can be effective Hammett parameters—experimentally determined values for a range of carboxylic acids—we envision that our descriptors would be similarly broadly applicable beyond the specific chemistry of carboxylic acids (*e.g.*, carboxylic acid descriptors can be used as a proxy for other carbonyl compounds like Hammett descriptors have been used for boronic acids,^68^ manganese-salen catalysts,^69^ anhydrides/acid chlorides^70^). Ultimately, this study provides a blueprint not only for descriptor library building but also for expanding those libraries to myriad related compounds using 2D and 3D GNN models. Taken together, these advances represent a step towards the overall effort to reduce the computational cost and lower the barrier of entry to perform data-driven chemical campaigns.

## Data availability

Computed descriptor libraries, GNN models, and summary statistics presented in this work are available on FigShare (https://doi.org/10.6084/m9.figshare.25213742.v3). Supporting code is available on Github (https://github.com/nsf-c-cas/AcidAmine_Descriptor_Predict/). Additional details clarifying methods are available as ESI (PDF).†

## Author contributions

B. C. H and M. A. H. conducted DFT designed and executed the workflow for the construction of the descriptor libraries. S. S. S. V. constructed the 2D GNN models and evaluated outliers. K. A. constructed the 3D GNN models. B. C. H and M. A. H. conducted external validation of the descriptor prediction models and the evaluation of error for downstream applications. C. W. C., R. S. P., and M. S. S. advised the work. All authors approved the final manuscript.

## Conflicts of interest

There are no conflicts to declare.

## Supplementary Material

DD-004-D4DD00284A-s001
