# Supplementary material for: Rapid prediction of conformationally-dependent DFT-level descriptors using graph neural networks for carboxylic acids and alkyl amines
Source: Digit Discov. 2024 Nov 28;4(1):222–33. doi: 10.1039/d4dd00284a (PMC11626426; doi:10.1039/d4dd00284a)
Supplement: DD-004-D4DD00284A-s001 [file DD-004-D4DD00284A-s001.pdf]

Supplementary Information (SI) for Digital Discovery.
This journal is © The Royal Society of Chemistry 2024

**SUPPORTING INFORMATION**

# Rapid Prediction of Conformationally-Dependent DFT-Level Descriptors using Graph Neural Networks for Carboxylic Acids and Alkyl Amines

Brittany C. Haas[1,†], Melissa A. Hardy[1,†], Shree Sowndarya S. V.[2,#], Keir Adams[3,#], Connor W. Coley[3,4,*], Robert S. Paton[2,*], Matthew S. Sigman[1,*]

*[1]Department of Chemistry, University of Utah, Salt Lake City, Utah 84112, United States*

*[2]Department of Chemistry, Colorado State University, Fort Collins, Colorado 80523, United States*

*[3]Department of Chemical Engineering, Massachusetts Institute of Technology, Cambridge, Massachusetts 02139, United States*

*[4]Department of Electrical Engineering and Computer Science, Massachusetts Institute of Technology, Cambridge, Massachusetts 02139, United States*

*[†]These authors contributed equally to this work.*

*[#]These authors contributed equally to this work.*

**Table of Contents**

1. Library Building .......... 3
  1.1 Molecule Identification .......... 3
    1.1.1 Reaxys Exports .......... 3
    1.1.2 Fragments from the Drug Repurposing Hub .......... 4
    1.1.3 Enamine Building Blocks Catalog .......... 5
  1.2 SMILES to .sdf Files .......... 5
  1.3 Conformational Searching & Conformer Selection .......... 5
  1.4 DFT Computations .......... 5
  1.5 Get Properties Notebook .......... 6
    1.5.1 Atom Map Preparation .......... 6
    1.5.2 Property Collection .......... 6
    1.5.3 Property Processing .......... 7
2. Considerations for Descriptor Prediction .......... 7
  2.1 Descriptors Predicted using 2D and 3D GNN Models .......... 7
  2.2 Descriptor Distribution .......... 8
  2.3 Atom Types in the Libraries .......... 14
  2.4 Investigating the Conformational Dependence of Properties .......... 15
3. Representations of Chemical Space .......... 15
  3.1 Descriptor Selection .......... 15

3.2 Principal Component Analysis .......... 16
3.3 Uniform Manifold Approximation Projections .......... 16
3.4 Combined Amine Library .......... 16
4. 2D GNN Models .......... 19
4.1 Overview of 2D Model .......... 19
4.2 Chemical Features Used to Represent Atoms and Bonds in Acids and Amines .......... 20
4.3 Analysis of Nearest Neighbors for Carboxylic Acid $H^5$ NMR Outlier .......... 21
4.4 2D-Based Gradient Boosting Regressor Models .......... 21
5. 3D GNN Models .......... 22
5.1 Overview of 3D Model .......... 22
5.2 Training Methods .......... 24
5.3 Analysis of 3D Model Performance on Outliers for the 2D model .......... 25
5.4 Comparing the Absolute Accuracies ($R^2$) of the 2D and 3D GNNs .......... 26
6. Evaluation of Acceptable Error .......... 28
6.1 Models Trained with DFT, 2D, and 3D GNN Predicted .......... 28
6.2 Generating Amide Couplings for Virtual Screening .......... 30
6.3 Comparing ln(*k*) Predictions from using DFT, 2D, and 3D GNN Predicted Descriptors .......... 30
6.4 Comparing ln(*k*) Predictions in Different Rate Regimes .......... 31
6.5 Acetic Acid as an Outlier .......... 31
6. SI References .......... 34

## 1. Library Building

An automated computational workflow to calculate DFT-derived molecular descriptors from SMILES string inputs was established for this work.

### 1.1 Molecule Identification

#### 1.1.1 Reaxys Exports

Using the Reaxys database, lists of carboxylic acids, primary alkyl amines, and secondary alkyl amines were identified via substructure searching and subsequent filtering of results.

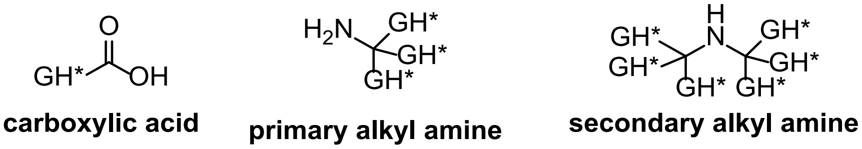


**Figure S1.** Carboxylic acid, primary alkyl amine, and secondary alkyl amine substructures employed in Reaxys searches. Per the Reaxys web interface, GH* is defined as “any group (+ ring closure) or H”.

*Carboxylic Acid Filtering*

Complete results of the substructure search (**Figure S1**) were first limited to those listed as “product for purchase” that also appeared in the Sigma-Aldrich database, in order to maintain a reasonable number of commercially available compounds. The results from this substructure search were further filtered to limit the results to compounds for which the MW < 504 g/mol (to avoid large peptides) and the number of fragments = 1 (to eliminate salts). Incompatible functional groups (alcohols, 1,2-amino alcohols, enols, primary amines, secondary amines, quaternary ammonium derivatives, α-amino acids, enamines, hydroxylamines, hemiaminals, thiohemiaminals, thiols, aryl thiols, boronic acids, sulfonic acids, phosphonic acids, and di-carboxylic acids) were explicitly excluded, and the results of the search were exported as a list of SMILES strings and numbered as Ac1–Ac8676.[a] Isotopically labeled acids (containing e.g., $^{2}H$, $^{13}C$, or $^{15}N$) were removed. In cases where the library contained racemic and enantiopure forms of the same acid, a single example was retained.

*Primary Amine Filtering*

Complete results of the substructure search (**Figure S1**) were first limited to those listed as “product for purchase” that also appeared in the Sigma-Aldrich database, in order to maintain a reasonable number of commercially available compounds. The results from this substructure search were further filtered to limit the results to compounds for which the MW < 504 g/mol (to avoid large peptides) and the number of fragments = 1 (to eliminate salts). Incompatible functional groups (carboxylic acids, amino acids, carboxylates, hydoxamic acids, secondary amines, quaternary ammonium derivatives, α-amino acids,

[a] Carboxylic acid searches were performed September 9th, 2021.

hydroxylamines, hemiaminals, thiohemiaminals, thiols, aryl thiols, alcohols, boronic acids, sulfonic acids, phosphonic acids, and di-amines) were explicitly excluded, and the results of the search were exported as a list of SMILES strings and numbered as N1–N4654.[b] Isotopically labeled amines (containing e.g., $^{2}$H, $^{13}$C, or $^{15}$N) were removed. In cases where the library contained racemic and enantiopure forms of the same amine, a single example was retained.

*Secondary Amine Filtering*

Complete results of the substructure search (**Figure S1**) were first limited to those listed as “commercial substrances”, in order to maintain a reasonable number of commercially available compounds. The results from this substructure search were further filtered to limit the results to compounds for which the MW < 504 g/mol (to avoid large peptides) and the number of fragments = 1 (to eliminate salts). Incompatible functional groups (carboxylic acids, amino acids, carboxylates, hydoxamic acids, carbamic acids, primary amines, quaternary ammonium derivatives, α-amino acids, hydroxylamines, hemiaminals, thiohemiaminals, thiols, aryl thiols, alcohols, boronic acids, sulfonic acids, phosphonic acids, and di-amines) were explicitly excluded. This left a list of over 88,000 results which were further limited to those that were “present as a reactant” and appeared in > 10 references; the results of the search were exported as a list of SMILES strings and numbered as SecN1–SecN4256.[c] Isotopically labeled amines (containing e.g., $^{2}$H, $^{13}$C, or $^{15}$N) were removed. In cases where the library contained racemic and enantiopure forms of the same amine, a single example was retained.

### 1.1.2 Fragments from the Drug Repurposing Hub

The Broad Institute’s Drug Repurposing Hub of FDA-approved, clinical trial, and pre-clinical trial drugs was queried for amide containing drugs.[1] The SMARTS string “CC(NC)=O” was used to identify drugs containing secondary and tertiary amides to obtain a list of 1988 compounds, 1744 of which could be handled by RDKit.[2,d] SMARTS filtering was used to eliminate drugs containing functional groups incompatible with an amide coupling reaction as described above (e.g., alcohols, thiols, etc.). Additionally, drugs containing multiple amides or amides that would fragment to give aryl or heteroaryl amines were excluded. RDKit was used to generate SMILES strings for the acid and amine fragments for each remaining amide. Acids, primary amines, or secondary amines already present in their respective libraries were removed.

---

[b] Primary alkyl amine search was performed October 11th, 2021, with “GH” instead of “GH*” groups. Per the Reaxys web interface, “GH” is defined as “any group or H”. The search was re-run on June 16th, 2022, with “GH*” and new results were added into the library.

[c] Secondary alkyl amine search was performed September 21st, 2022.

[d] Search of the Broad Institute’s Drug Repurposing Hub was performed August 25th, 2023.

### 1.1.3 Enamine Building Blocks Catalog

Enamine's top 50 carboxylic acid, primary amine, and secondary amine building blocks lists were downloaded.[e] SMARTS filtering was used to eliminate compounds containing functional groups incompatible with an amide coupling reaction as described above (e.g., alcohols, thiols, etc.). Compounds that were listed as salts were calculated as the neutral species. Acids, primary amines, or secondary amines already present in their respective libraries or generated from the Drug Repurposing Hub were removed.

## 1.2 SMILES to .sdf Files

SMILES strings were converted to Structure-Data Files (.sdf) using RDKit.[2] Strings that failed to produce an .sdf or produced an incorrect structure (i.e., open-shelled) with RDKit were attempted with Open Babel. In cases where Open Babel also failed to produce a correct .sdf, the SMILES string was used to build the molecule directly in Schrödinger Maestro's[3] 2D sketcher.

## 1.3 Conformational Searching & Conformer Selection

To accurately capture the conformational flexibility of substrates at a reasonable computational cost, a conformational searching and conformer selection procedure was implemented. Each compound (carboxylic acids and amines) was subject to a molecular mechanics conformational search using Schrödinger MacroModel[2] and the OPLS4 force field.[4] MacroModel conformational searches were run in the gas phase, with a maximum of 10,000 interactions, and a convergence threshold of 0.001. An ensemble of conformers within 21 kJ/mol (5.02 kcal/mol) of the minimum were collected, excluding mirror-image conformers. If the ensemble consisted of greater than 20 conformers, the number of conformers was reduced by atomic root mean square deviation (RMSD)-clustering to the minimum Kelley penalty value[5] and taking the centroids of the resultant clusters. The Kelley index facilitates selection of the optimal number of clusters and ensures the selected conformers structurally diverse.

## 1.4 DFT Computations

All quantum mechanical (DFT) geometry optimization and single point calculations were performed using Gaussian 16 (revision C.01).[6] Geometry optimizations and sequent frequency calculations of the selected conformers were performed at the B3LYP[7]-D3(BJ)[8]/6-31G(d,p)[9]-LANL2DZ(I, Sn, Se)[10] level of theory with cartesian d functions up to 6D with an ultrafine integration grid and root mean square (RMS) force criterion of $3 \times 10^{-4}$. All optimized geometries were verified by frequency computations as minima (zero imaginary frequencies). The resultant geometries were subject to single point calculations at the M06-2X[11]/def2-TZVP[12]-SDD(I, Sn, Se)[13] level of theory. Determination of the natural bond orbitals (NBO) and

---

[e] Enamine's top 50 carboxylic acid, primary amine, and secondary amine building blocks were exported from https://enamine.net/building-blocks/functional-classes on August 25th, 2023.

natural population analysis (NPA) was performed using the NBO program (version 7.0).[14] NMR shifts were computed using the gauge-invariant atomic orbitals (GIAO) method.[15]

For conformers where the optimization calculation did not terminate normally or the frequency calculations resulted in an imaginary frequency, the conformer was resubmitted specifying tight convergence criteria (using Gaussian keyword opt=tight) and the calculation of force constants (using Gaussian keyword opt=calcfc).[4] Several attempts were made; however, if a job could not terminate normally or an optimized structure devoid of any negative frequencies could not be obtained for a given conformer, that conformer was removed from the ensemble for descriptor collection. In a few cases, a conformer optimized to a geometry that could not be handled by RDKit[2] and produced irregular valences (e.g., due to intramolecular coordination between an amine and a borinic ester) these conformers were removed from the ensemble for descriptor collection.

### 1.5 Get Properties Notebook

A Jupyter Notebook was designed to collect automate the collection of molecular properties, as well as atom- and bond-level properties for a conserved moiety of interest from Gaussian jobs. Post-processing allows for the collection of condensed descriptors for conformational ensembles. Code adaptable to any moiety of is available on GitHub (https://github.com/SigmanGroup/Get_Properties).[16]

#### 1.5.1 Atom Map Preparation

RDKit[2] was used to extract and tabulate the atom numbers of structural motifs specified by a SMARTS string. The carboxylic acid, primary alkyl amine, and secondary alkyl amine SMARTs strings as well as the general atom labels used for molecular descriptor collection are shown in **Figure S2**.

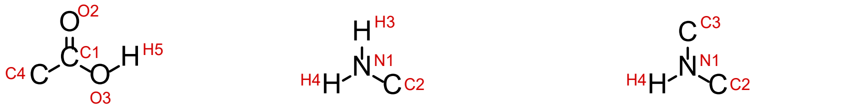


**carboxylic acid** [*]C(O[H])=O

**primary alkyl amine** [H]N([*])[H]

**secondary alkyl amine** [H]N([*])[*]

**Figure S2.** Atom numbering used for property collection.

In a few cases (<0.005%), RDKit[2] could not parse the structure and atom maps were generated manually.

#### 1.5.2 Property Collection

Properties allow for quantification of a structure's steric, electronic, and stereoelectronic features. Code specific to the properties collected for the carboxylic acid, primary alkyl amine, and secondary alkyl amine libraries is available on GitHub (https://github.com/nsf-c-cas/AcidAmine_Descriptor_Predict/). Complete

databases of collected properties can be found on Figshare (https://doi.org/10.6084/m9.figshare.25213742.v3).

### 1.5.3 Property Processing

The properties collected for each conformer were used to determine a set of descriptors for each molecule. *Properties* are ascribed to the individual conformers. *Descriptors* are used to describe the molecule itself and are the result of properties from a molecule's conformational ensemble.
The Boltzmann-weighted average (Boltz) was calculated using the relative free energies corrected with the electronic energy from the gas phase single points. Of note, is that this is only an estimate of the actual Boltzmann-weighted average descriptors because an incomplete, but representative, set of conformers is used when clustering is required (described above).

**Table S1.** Descriptors collected for each molecule.

| Descriptors | Definition |
|---|---|
| **Boltz** | Boltzmann-weighted average of a property from all the conformers in an ensemble (T = 298.15 K) $$Boltz\ avg\ property = \sum^{all\ confs} \frac{e^{-\Delta G_i / RT}}{\sum^{all\ confs} e^{-\Delta G_i / RT}} \cdot property_i$$ |
| **max** | highest value of a property given by any conformer in an ensemble |
| **min** | lowest value of a property given by any conformer in an ensemble |
| **low_E** | value of a property from the lowest energy conformer in the ensemble |
| **Boltz_stdev** | Boltzmann-weighted (weighted by mole fraction) standard deviation of a property based on all the conformers in an ensemble (T = 298.15 K) $$Boltz\ stdev = \sqrt{\frac{\sum_{i=1}^{N} \omega_i\,(x_i - \bar{x}^*)^2}{\frac{(N-1)}{N}\sum_{i=1}^{N}\omega_i}}$$ $N$ = total number of conformers<br>$\omega_i$ = mole fraction<br>$x_i$ = property value for each conformer<br>$\bar{x}$ = weighted mean of the property |

## 2. Considerations for Descriptor Prediction

### 2.1 Descriptors Predicted using 2D and 3D GNN Models

A subset of descriptors collected in each library were selected for prediction with GNN models. For all property types, Boltzmann-weighted standard deviation descriptors were excluded. Additionally, we did not predict derived properties (e.g., η, μ, ω, sphericity) or geometric properties (e.g., atom distances and

angles) able to be extracted directly from a graph representation. In the case of buried volume, the descriptor was calculated at several radii, but the descriptor at a single radius was selected to predict for each library. Complete lists of descriptors included in each library, predicted using 2D GNN model, and predicted using 3D GNN models is available in 2D_and_3D_test_and_ext_val_statistics.xlsx (https://doi.org/10.6084/m9.figshare.25213742.v3).

### 2.2 Descriptor Distribution

The distribution of each descriptor collected are plotted below (**Figure S3-S8**). A normal distribution is exhibited across different property types (e.g., molecule-, bond-, atom-level), descriptors (e.g., Boltz, max, min, low_E), and libraries (e.g., carboxylic acid, primary alkyl amine, and combined alkyl amine).

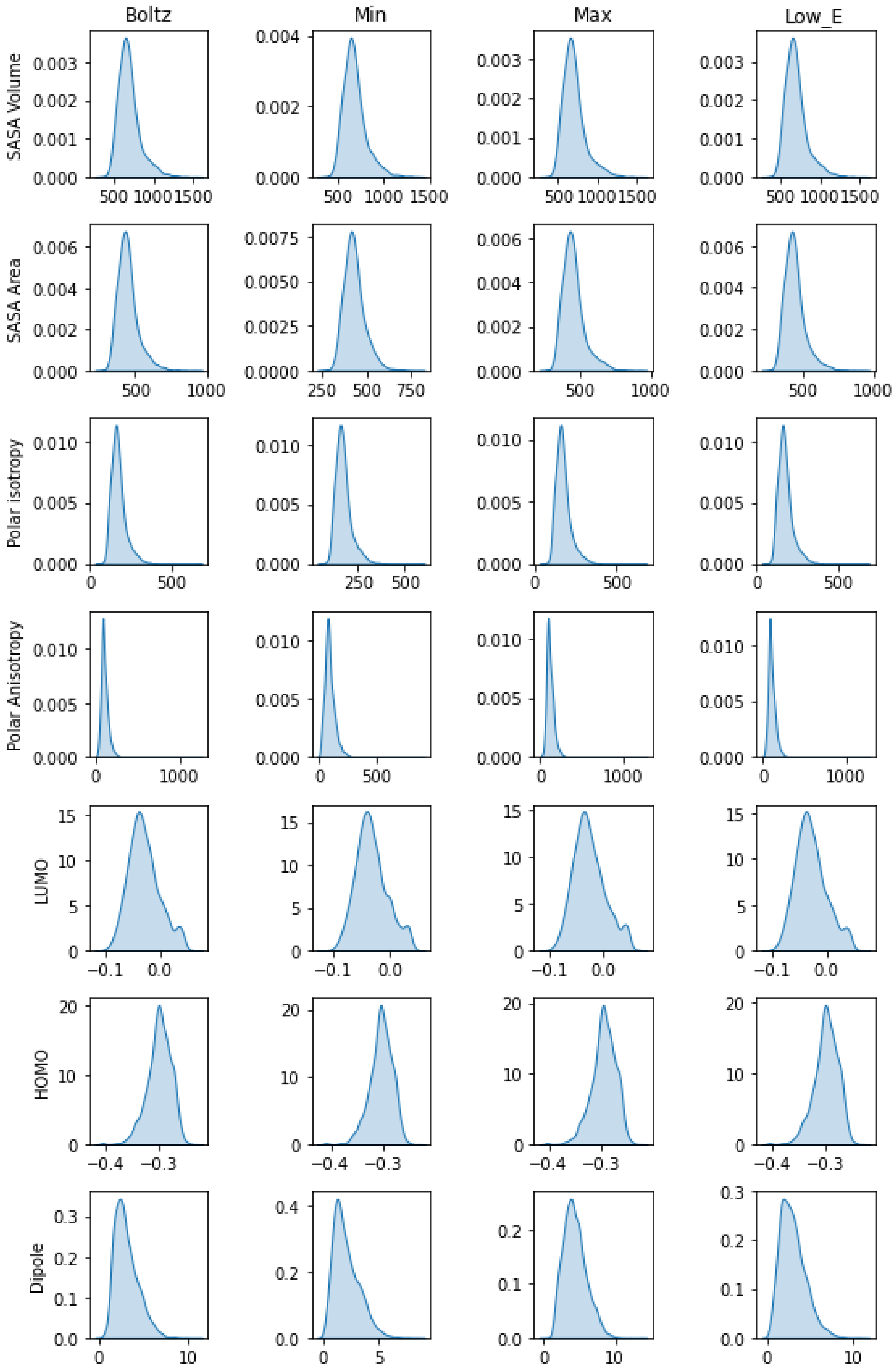


**Figure S3.** Distribution of molecule-level descriptors for the carboxylic acid library.

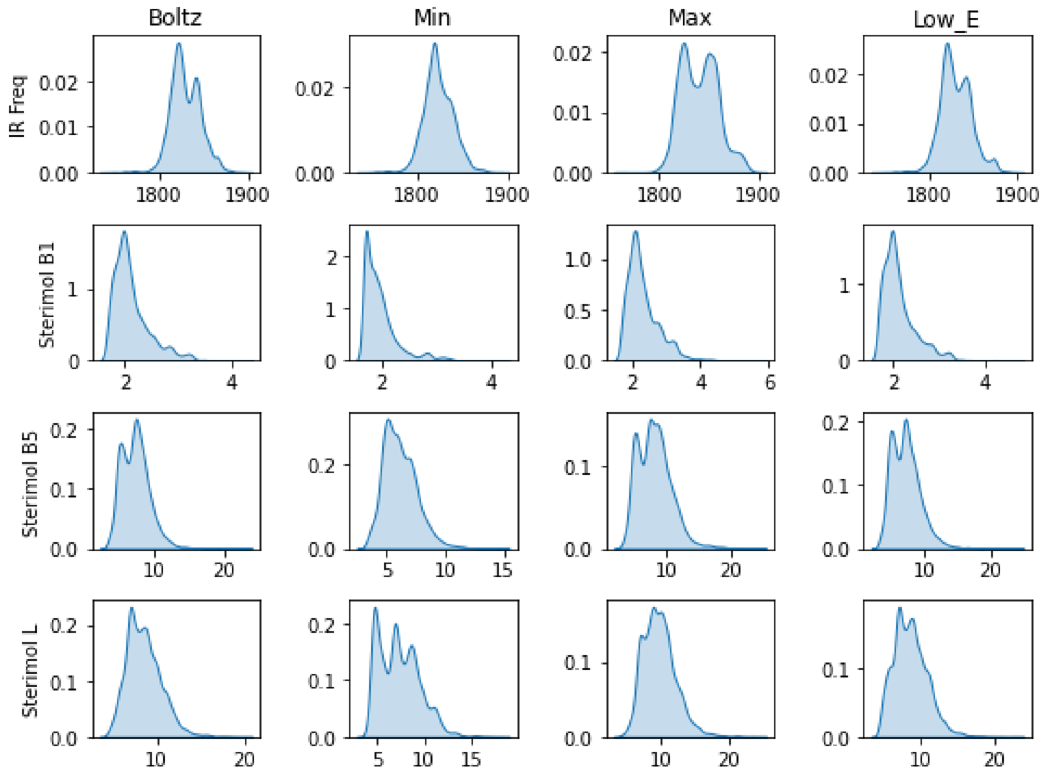


**Figure S4.** Distribution of bond-level descriptors for the carboxylic acid library.

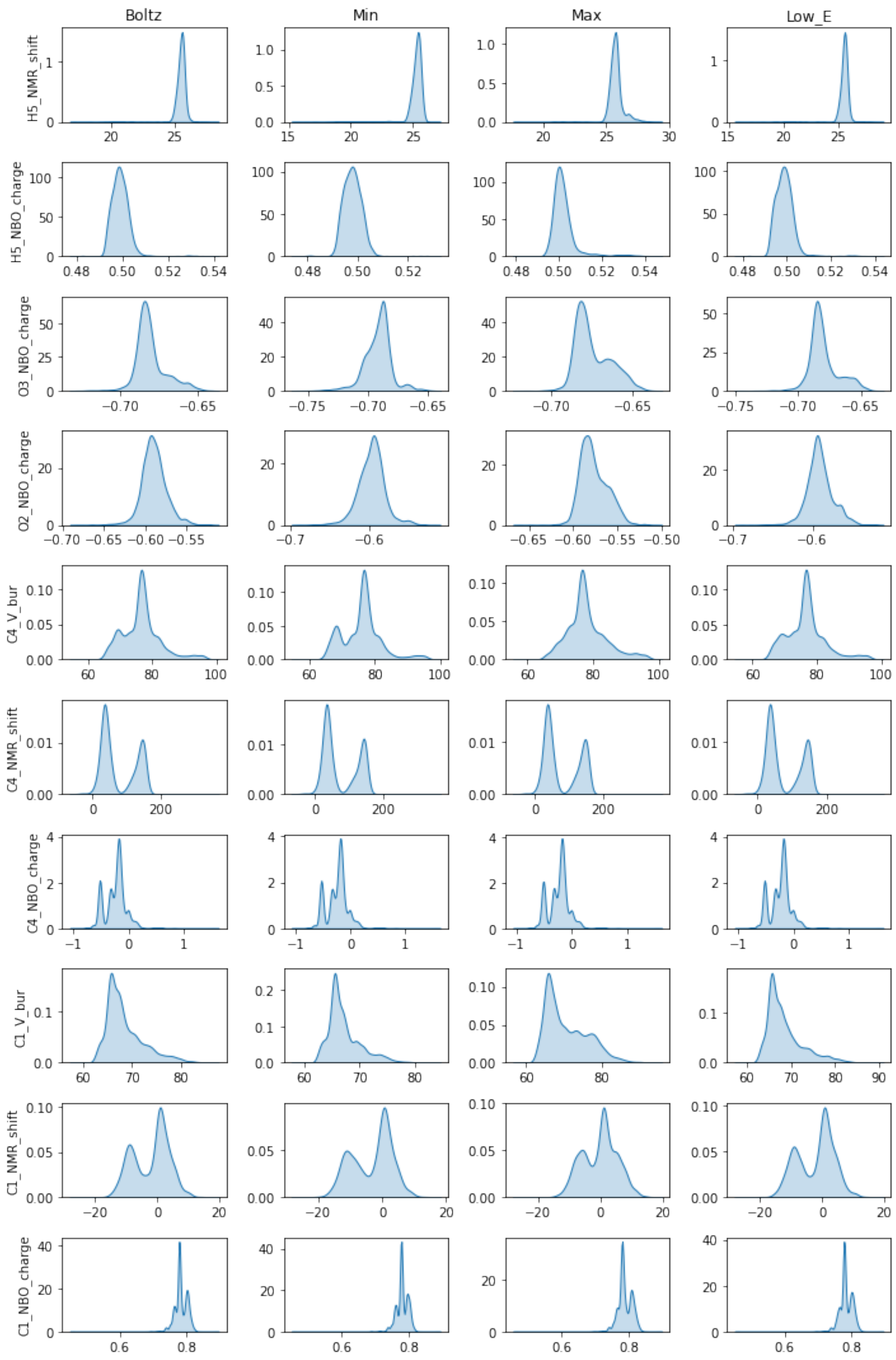


**Figure S5.** Distribution of atom-level descriptors for the carboxylic acid library.

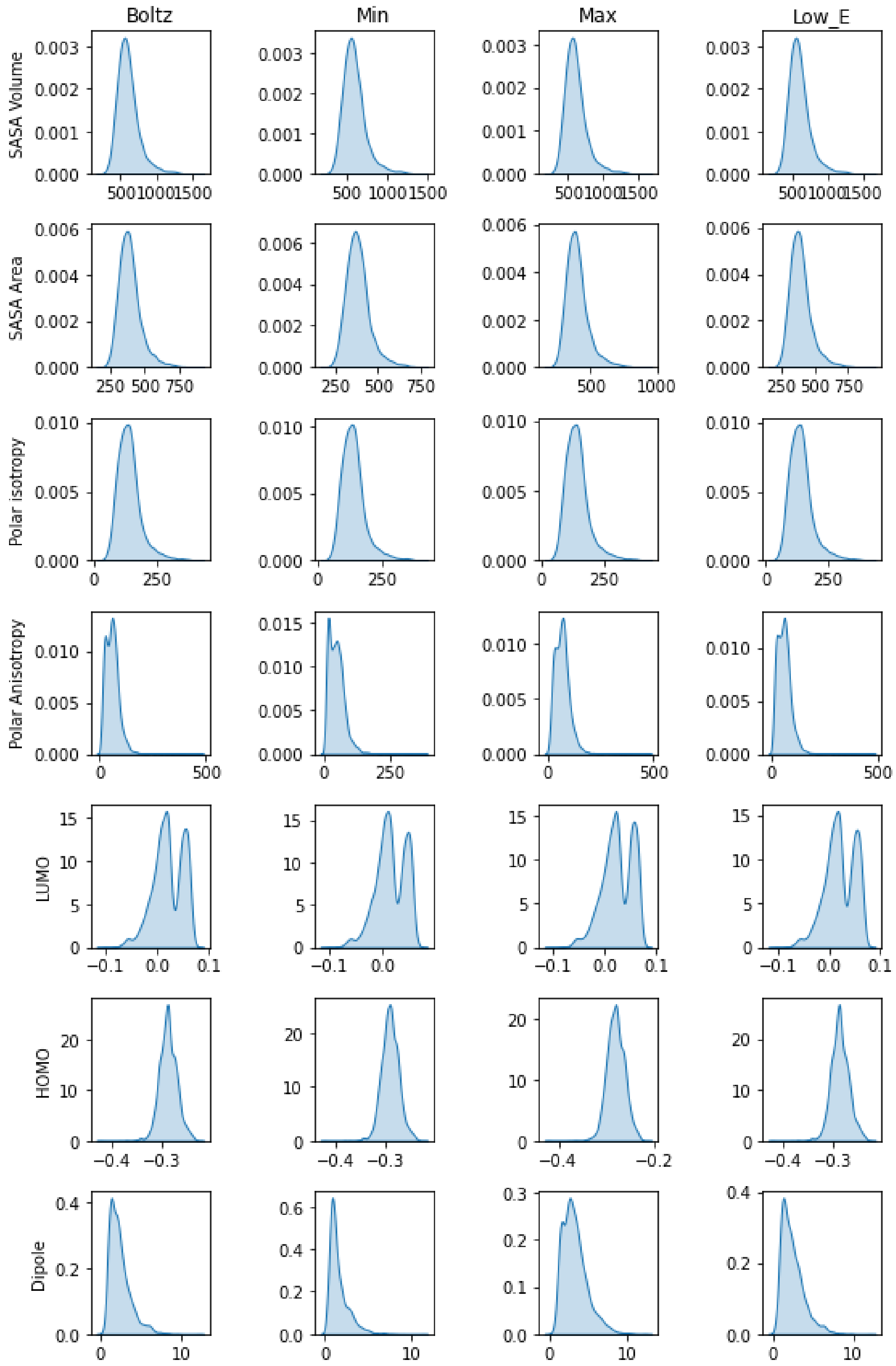


**Figure S6.** Distribution of molecule-level descriptors for the combined alkyl amine library.

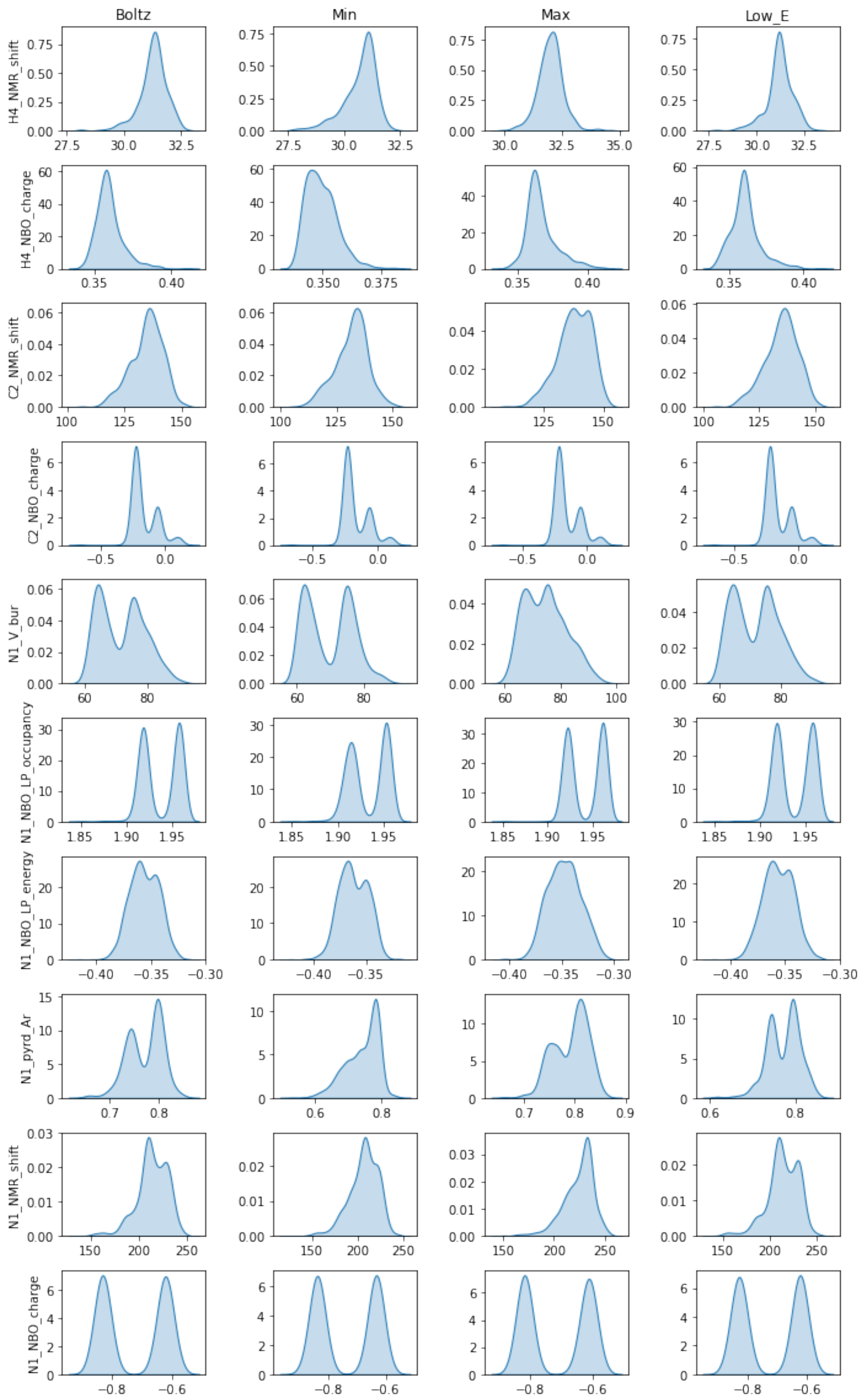


**Figure S7.** Distribution of atom-level descriptors for combined alkyl amine library. $N^1$ properties correspond to the combined library of primary and secondary alkyl amines. $C^2$ and $H^4$ properties are for primary and secondary alkyl amines, respectively.

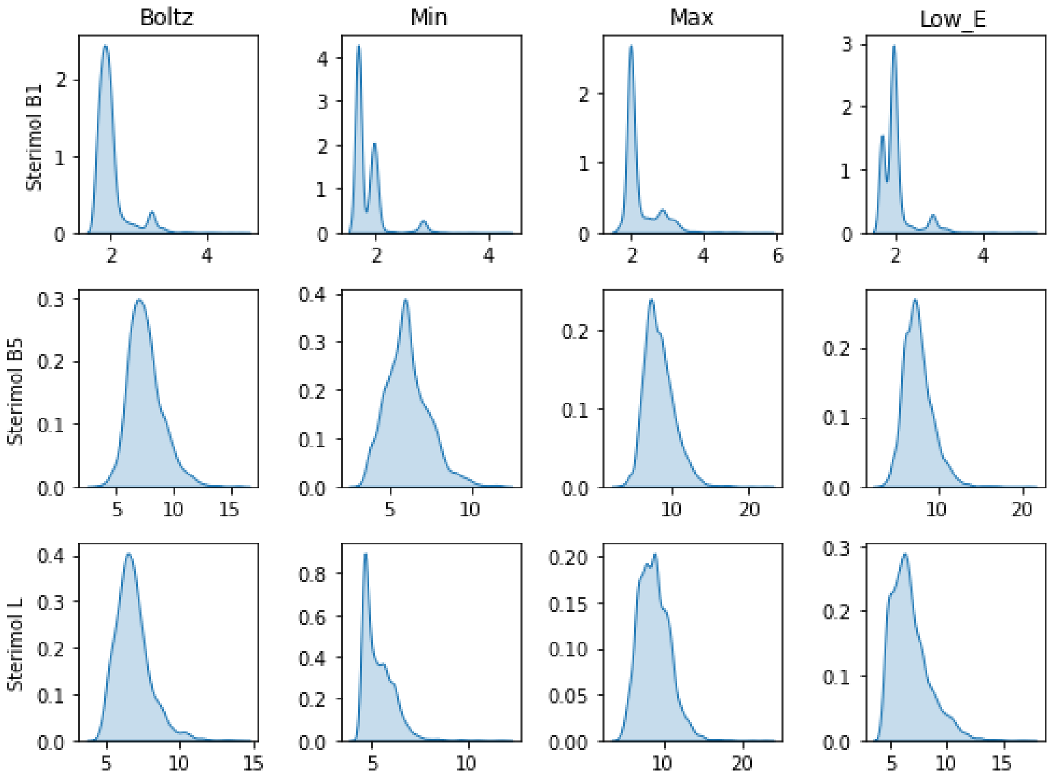


**Figure S8.** Distribution of bond-level descriptors for primary alkyl amine library.

### 2.3 Atom Types in the Libraries

**Table S2.** Atoms counts in the carboxylic acid and combined alkyl amine libraries.

| Atom Type | Carboxylic Acids | Combined Alkyl Amines |
|---|---|---|
| C | 101925.0 | 83368.0 |
| O | 26389.0 | 7143.0 |
| H | 99810.0 | 126067.0 |
| N | 6215.0 | 13099.0 |
| F | 4271.0 | 2297.0 |
| S | 1933.0 | 692.0 |
| Cl | 1931.0 | 732.0 |
| Br | 1540.0 | 351.0 |
| I | 268.0 | 23.0 |
| P | 13.0 | 55.0 |
| B | 60.0 | 23.0 |
| Si | 23.0 | 114.0 |
| Sn | - | 11.0 |
| Se | - | 1.0 |

## 2.4 Investigating the Conformational Dependence of Properties

We hypothesized that the conformational dependence was more accentuated of some properties (e.g., Sterimol L, $B_1$ and $B_5$) than other (e.g., HOMO and LUMO). The conformational dependence was quantified by equation **S1**.

$$conformational\ dependence = $$
$$mean\ or\ median\left(\frac{stdev(conformer\ property)_{a\ conformer\ ensemble}}{stdev[mean(conformer\ properties)_{a\ conformer\ ensemble}]_{all\ molecules\ \ in\ library}}\right)$$

(**Equation S1**)

Conformational dependence as determined by the mean or median of all molecules in a library are plotted in **Figure S9**. These results are consistent with our findings that the highly conformationally dependent descriptors required 3D GNN models to obtain high fidelity predictions.

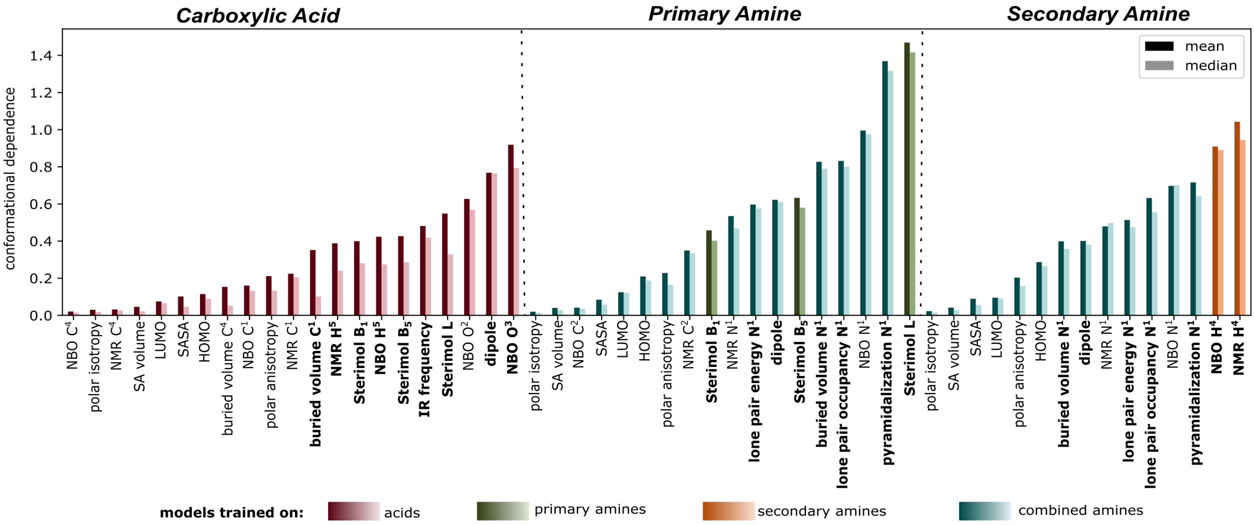


**Figure S9.** Bar graph showing the conformational dependence as determined by the mean and median of all molecules in the respective libraries. Bar colors indicate the dataset that the descriptor model was trained on. 3D GNN models were trained for properties with bolded labels.

## 3. Representations of Chemical Space

Chemical space projections for each library were generated by applying dimensionality reduction techniques to a curated set of Boltzmann-averaged descriptors.

## 3.1 Descriptor Selection

From the Boltzmann-averaged descriptors in each of the carboxylic acid, primary alkyl amine, and secondary alkyl amine libraries, a curated set was down-selected to exclude colinear descriptors ($R^2$ > 0.95) and descriptors that are linear combinations of others in the library (e.g., η, μ, ω, sphericity). The

curated set of descriptors from each library is available in the “Descriptors_PCA_and_UMAP” sheet in each library file (https://doi.org/10.6084/m9.figshare.25213742.v3).

### 3.2 Principal Component Analysis

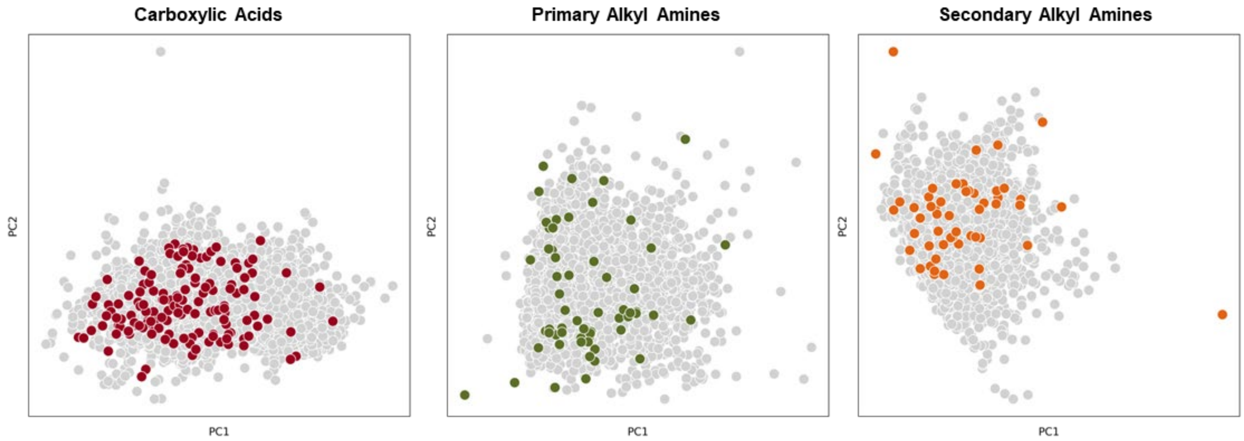


**Figure S10.** PCA plots of carboxylic acids, primary alkyl amines, and secondary alkyl amines.

Principle component analysis (PCA)[17] was applied to the curated set of descriptors for each of the carboxylic acid, primary alkyl amine, and secondary alkyl amine libraries (**Figure S10**). In each case, enough PCs were generated to explain a total of 75% of the variance in the dataset (3 to 7 PCs). The relative contribution of each descriptor to each PC is available in the “PCA_Contributions” sheet in each library (https://doi.org/10.6084/m9.figshare.25213742.v3). The PC coordinates of each compound are also available in the “PCA_and_UMAP_Coordinates” sheet in each library (https://doi.org/10.6084/m9.figshare.25213742.v3)

### 3.3 Uniform Manifold Approximation Projections

The same curated set of descriptors was processed using a uniform manifold approximation projection (UMAP) (as shown in Figure 2).[18] The UMAP coordinates of each compound are available in the “PCA_and_UMAP_Coordinates” sheet in each library (https://doi.org/10.6084/m9.figshare.25213742.v3).

### 3.4 Combined Amine Library

Given the overlap in the feature space of primary alkyl amines and secondary alkyl amines, we generated a combined library of amine descriptors and corresponding chemical space projections (**Figure S11**). It is noted that primary and secondary amines populate distinct areas of our projected chemical space.

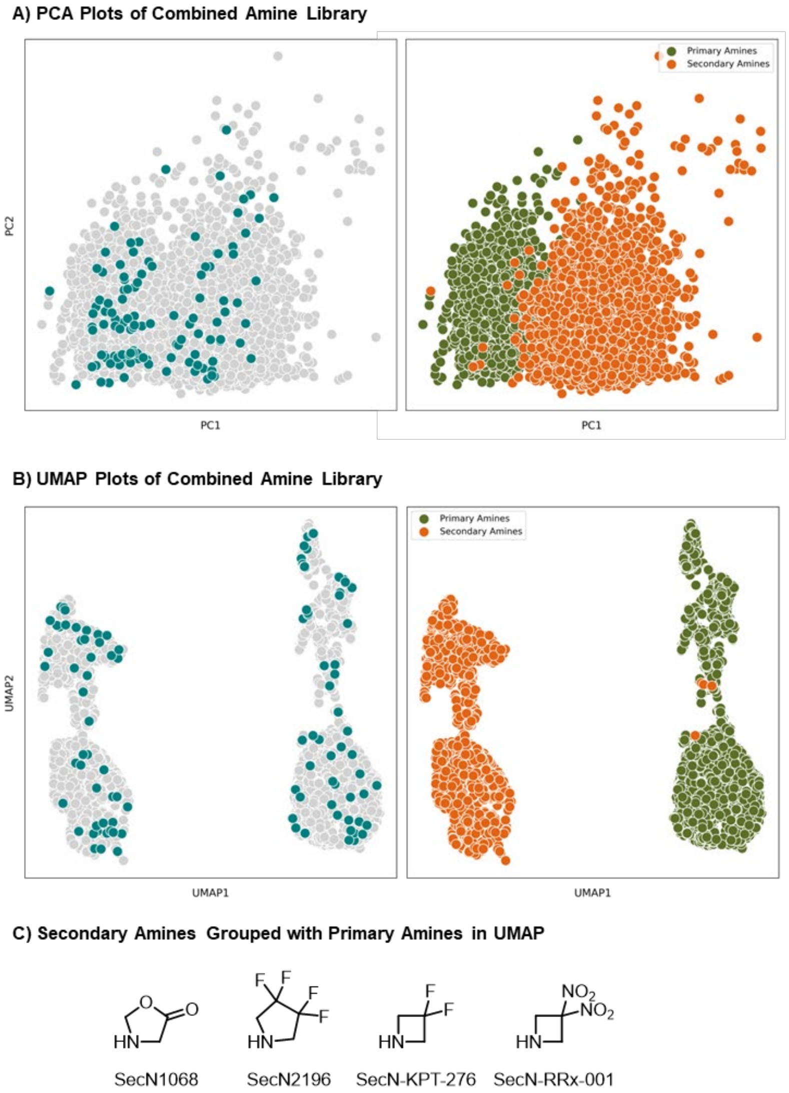


**Figure S11.** Chemical space representations of combined amine library. (A) PCA plot of combined amine library with the external validation set highlighted (left) or the primary and secondary amines color coded (right). (B) UMAP plot of combined amine library with the external validation set highlighted (left) or the primary and secondary amines color coded (right). (C) Chemical structures of the four secondary amines that are closer to the primary amines in the UMAP chemical space projection.

For each descriptor, we analyzed the distribution of primary and secondary amines. Generally, molecular descriptors had overlap between the two subclasses (**Figure S12A**); however, atom-level descriptors (**Figure S12B**) typically had little overlap in descriptor values with the most extreme example being the Boltzmann-average NBO partial charge of $N^1$.

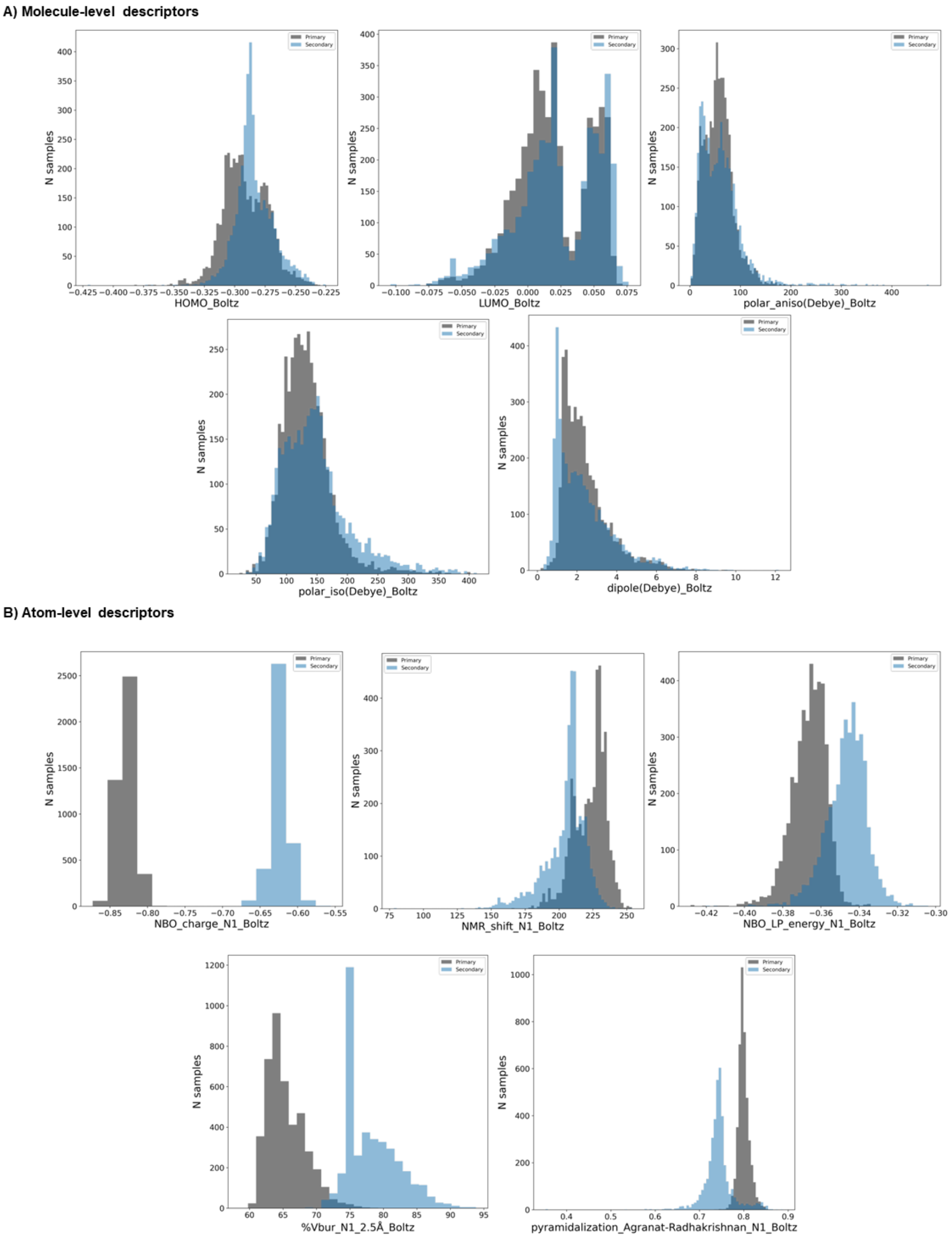


**Figure S12.** Histograms showing the distribution of primary and secondary alkyl amine descriptor values.

## 4. 2D GNN Models

### 4.1 Overview of 2D Model

The architecture for modeling carboxylic acid and alkyl amine descriptors can be broken down into three parts: namely, node and edge featurization, model block for training, and descriptor prediction. The first step involved the conversion of the carboxylic acid or alkyl amine to graphs with chemical information as detailed below. This was performed for the train/validation/test subsets on-the-fly when creating batches of size 128 for property training. Subsequently, training with a learning rate of 0.0001 for 1000 epochs using mean squared error as the loss function. The training model (model block) consists of the following layers: 1) an encoding layer that linearly expands the dimension of the atom and bond features to 128, 2) the message-passing block utilizing GINE convolutions to pass information between nodes of the graph, and 3) a final linear layer followed by a swish activation layer to gain updated node representations for graphs representing carboxylic acids or amines. Upon gaining these updated representations for nodes, three different pooling techniques are utilized for molecule-, atom-, and bond-level properties. For the molecular properties, the sum of all node representations is used ($h_{agg}$). The atomic properties are predicted for the respective atom (i.e., C1, O2, O3, C4, H4 in carboxylic acids and N1, C2, H4 in amines) by picking the corresponding node representation ($h_i$) and concatenating it with the mean representation ($h_{agg}$) of all nodes. The bond properties are predicted for the respective bond (i.e., C1, O2 in carboxylic acids and N1, C2 in primary amines) by picking nodes representation in the corresponding bonds, summing them ($h_{ij}$ and $h_{ji}$), then concatenating the summed representation with the mean representation ($h_{agg}$) of all nodes. The formulation of how equations is shown in **Figure S13.**

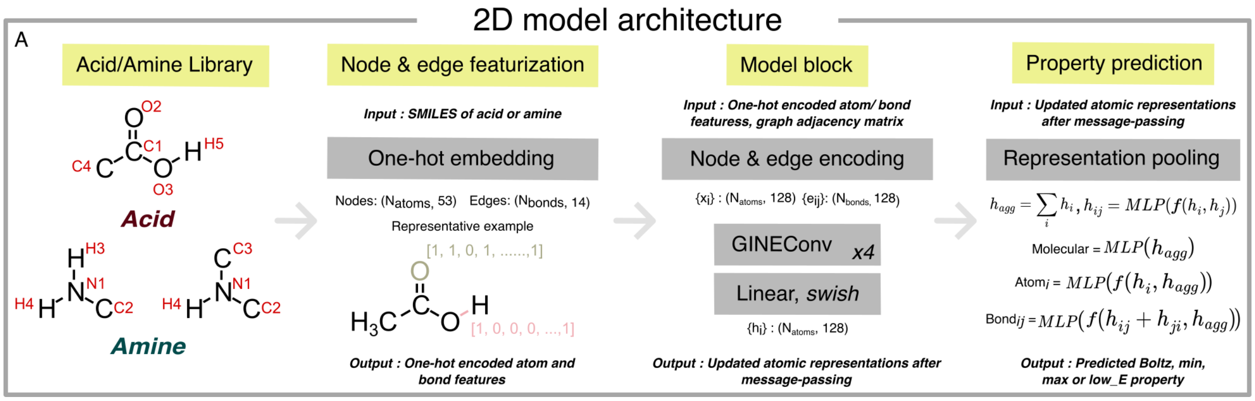


**Figure S13.** Depiction of the 2D model architecture for all properties with a breakdown of differences for molecular, atomic, and bond properties.

Training, validation, and test splits[f] and all code associated with the models published herein is available as 2D.zip (https://doi.org/10.6084/m9.figshare.25213742.v3) and on GitHub (https://github.com/nsf-c-cas/AcidAmine_Descriptor_Predict/).

## 4.2 Chemical Features Used to Represent Atoms and Bonds in Acids and Amines

Cheminformatics toolkit RDKit[2] was used to featurize the carboxylic acid and alkyl amine libraries for developing neural network models. The list of features for atoms and bonds is shown in **Table S3**. Each atom/bond property was one-hot encoded based on the list of features. Upon one-hot encoding, each atom and bond were represented as a vector of dimension (1, 53) and (1,14), respectively. When creating these representations for graphs of molecules, we only incorporated the hydrogen atoms involved in the substructure of carboxylic acid or amine functional group. Finally, upon converting a molecule to a graph structure with one-hot representations, they were utilized in training graph neural networks to predict carboxylic acid or alkyl amine descriptors.

**Table S3.** Atom and bond properties that were used for one-hot encoding.

| **Atom** | | **Bond** | |
|---|---|---|---|
| **Atom Symbol** | C, O, H, N, F, S, Cl, Br, I, P, B, SI, Sn. Se | **Bond type** | 'SINGLE', 'DOUBLE', 'TRIPLE', 'AROMATIC' |
| **Num. radical electrons** | 0, 1, 2 | **Is a conjugated bond** | True, False |
| **Chiral tag (RDKit based)** | CHI_UNSPECIFIED, CHI_TETRAHEDRAL_CW, CHI_TETRAHEDRAL_CCW, CHI_OTHER | **Is in a ring bond** | True, False |
| **Formal charge** | -1, -2, 1, 2, 0 | **Stereochemistry** | STEREONONE, STEREOANY, STEREOZ, STEREOE, STEREOCIS, STEREOTRANS |
| **Is aromatic atom** | True, False | | |
| **In a ring of size n** | 0,1,2,3,4,5,6 | | |
| **Degree** | 0, 1, 2, 3, 4, 5, 6 | | |
| **Total number of Hs** | 0,1,2,3,4,5,6 | | |

[f] Four compounds not retained in the final library were erroneously used in the training set for the 2D models. Two of these compounds (C[C@H]1CN(C(=O)OC(C)(C)C)CC[C@H]1C(=O)O and COc1ccc(CN2C[C@H](C(=O)O)CC2=O)c(OC)c1) had enantiomeric forms already in the library and the other two (C[Si](C(=O)O)(c1ccccc1)c1ccccc1 and CC(C)(C)OC(=O)N1CCN(C(=O)O)CC1) were not carboxylic acids.

### 4.3 Analysis of Nearest Neighbors for Carboxylic Acid $H^5$ NMR Outlier

The 2D GNN predicted Boltzmann averaged $H^5$ NMR shift of carboxylic acid **2** was 6.8 ppm larger than the DFT calculated descriptor. While we recognize that intramolecular hydrogen bonding should increase the NMR shift of the carboxylic acid hydrogen (downfield shift, experimental value >10 ppm), the predicted shift of 25.9 ppm was significantly higher than even the very high calculated shift of 19.1 ppm. To understand this further, nearest neighbor analysis identified the ten most similar substrates in the training data (**Figure S14**). This analysis utilizes the output (128-vector) of the final to last layer in the model defined in **Figure S13**. These latent vectors for the whole training database are calculated and are used to estimate the distance between the carboxylic acid **2** vector and the other carboxylic acids in the training set. Of note, the most similar molecules in the provided training data contain similar functional groups and their DFT calculated Boltzmann averaged $H^5$ NMR shifts are notably high (23-27 ppm, like the predicted value for **2**).

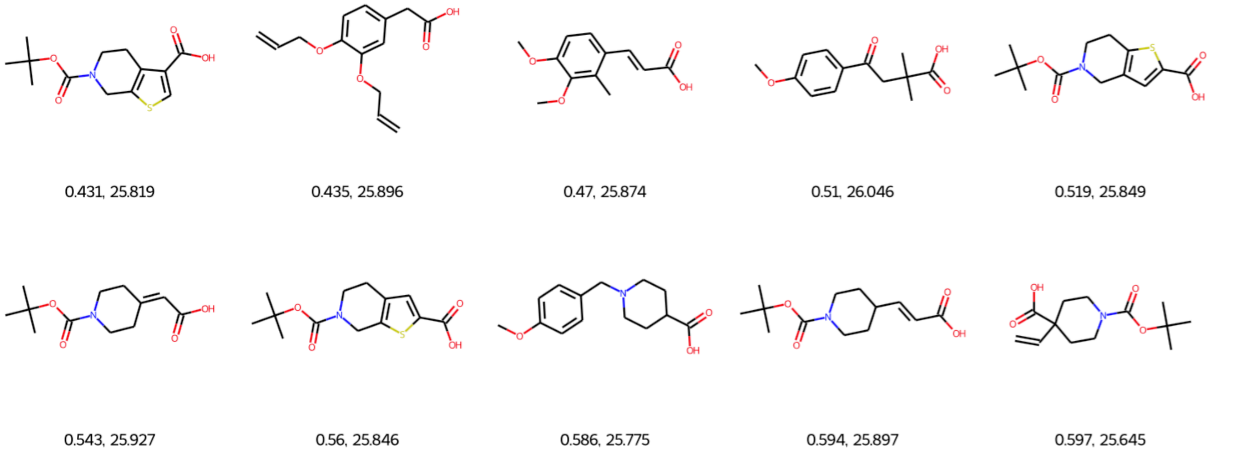


**Figure S14.** The ten nearest neighbors to carboxylic acid **2**, identified as an outlier in the prediction of Boltzmann averaged $H^5$ NMR shift. Below each molecule is the distance in latent vector chemical space (left) and the DFT calculated Boltzmann averaged $H^5$ NMR shift (right).

### 4.4 2D-Based Gradient Boosting Regressor Models

To validate the use of GNNs for descriptor predictions, we compared GNN vs gradient boosting regressor (GBR) models for representative molecule-, bond-, and atom-level carboxylic acid descriptors. First, the carboxylic acids were converted to molecular fingerprints with chemical information using the cheminformatics toolkit RDKit.2 Morgan fingerprints with a radius of 4 and a feature size of 512 bits were employed to gain numerical representations of the molecules from SMILES. This was performed with the same training/test sets used for the GNN models. Using the molecular fingerprints, a GBR model was trained using a random CV search to obtain the best hyperparameters for the model as implemented in scikit-learn. The final parameters were optimized individually for each carboxylic acid descriptor modeled: molecule descriptor (HOMO_Boltz), bond descriptor (Sterimol_L_C1_C4(Å)_morfeus_Boltz), and atom

descriptors (NMR_shift_C1_Boltz). A comparison of the GNN and GBR models is presented in **Table S4**. We note that the GNN model performs better than the fingerprint-based GBR model for the molecule, bond, and atom-level acid descriptors evaluated. This highlights that the GNN models are learning better representations (i.e., graphs vs. fingerprints) of the molecules resulting in higher accuracy.

**Table S4.** Comparison of 2D models using GNN and GBR for representative carboxylic acid descriptors.

| Acid Boltzmann-Averaged Descriptor | Descriptor Type | GNN Test $R^2$ | GNN Test MAE | GBR Test $R^2$ | GBR Test MAE |
|---|---|---|---|---|---|
| HOMO (Boltz) | molecule | 0.97 | 0.00 | 0.29 | 0.01 |
| Sterimol L (Boltz) | bond | 0.80 | 0.57 | 0.65 | 0.86 |
| NMR $C^1$ (Boltz) | atom | 0.97 | 0.56 | 0.83 | 1.66 |

## 5. 3D GNN Models

### 5.1 Overview of 3D Model

We employed DimeNet++ as the backbone architecture of the 3D GNNs but applied minor modifications to the atom embedding and readout layers of the network, as described below. For further technical details regarding the model architecture, we refer readers to the original papers introducing DimeNet/DimeNet++[19][20] as well as to the codebase.

As a standard E(3)-invariant 3D graph neural network, DimeNet++ encodes 3D molecular structures, where each structure with $N$ atoms is represented as an attributed point cloud $\boldsymbol{P} = \{(\boldsymbol{r}_i, \boldsymbol{x}_\mathrm{i})\}_{i=1}^{N}$ where $\boldsymbol{r}_i \in \mathbb{R}^3$ are the Euclidean coordinates of atom $i$ with features $\boldsymbol{x}_\mathrm{i} \in \mathbb{R}^{d_x}$. For any molecule-, bond-, or atom-level descriptor *not* involving a hydrogen atom, we only included non-hydrogen atoms in the point cloud. For atom-level descriptors of hydrogen atoms (e.g., $H^4$ NBO charge of secondary amines), we additionally include the hydrogen atoms of the acid or amine chemical moiety. Whereas the original implementation of DimeNet++ uses only one-hot representations of the element type as $\boldsymbol{x}_\mathrm{i}$, we used additional atom features as described in the 2D modeling section.

Given a structure $\boldsymbol{P}$, DimeNet++ uses geometry-informed message passing to encode $\{\boldsymbol{h}_i\}_{i=1}^{N} = f_{DimeNet++}(\boldsymbol{P})$, where $\boldsymbol{h}_i \in \mathbb{R}^{d_h}$ is the atom-specific representation of atom $i$. To adapt DimeNet++ to our supervised learning tasks, our models pool $\{\boldsymbol{h}_i\}_{i=1}^{N}$ into atom-, bond-, or molecule-level representations prior to predicting the scalar regression target $y$.

$$\boldsymbol{h}_{agg} = \sum_{i=1}^{N} \boldsymbol{h}_i$$

(**Equation S2**)

*For atom-level descriptors:*

$$\boldsymbol{h}_{atom} = (\boldsymbol{h}_a, \boldsymbol{h}_{\boldsymbol{agg}})$$
$$y_{atom} = MLP_{atom}(\boldsymbol{h}_{atom})$$

(**Equation S3**)

*For bond-level descriptors:*

$$\boldsymbol{h}_{bond} = MLP_{perm}(\boldsymbol{h}_a, \boldsymbol{h}_b) + MLP_{perm}(\boldsymbol{h}_b, \boldsymbol{h}_a)$$
$$y_{bond} = MLP_{bond}(\boldsymbol{h}_{bond})$$

(**Equation S4**)

*For molecule-level descriptors:*

$$\boldsymbol{h}_{mol} = \boldsymbol{h}_{agg}$$
$$y_{mol} = MLP_{mol}(\boldsymbol{h}_{mol})$$

(**Equation S5**)

Here, $MLP_{atom}$, $MLP_{bond}$, and $MLP_{mol}$ are multi-layer perceptrons that map the pooled representations to scalar outputs. $\boldsymbol{h}_{agg}$ is an aggregated (sum-pooled) representation of the global molecule structure. $\boldsymbol{h}_a \in \{\boldsymbol{h}_i\}_{i=1}^{N}$ and/or $\boldsymbol{h}_b \in \{\boldsymbol{h}_i\}_{i=1}^{N}$ are representations of user-specified atoms in the molecule; in the case of predicting bond-level descriptors, atoms $a$ and $b$ form a covalent bond. $MLP_{perm}$ is a multi-layer perceptron that encodes a pair of (covalently-bonded) atoms into a permutation-invariant representation.

We use one set of hyperparameters to train each individual DimeNet++ model. The default hyperparameters are listed in **Table S5**.

**Table S5.** Default hyperparameters used for all DimeNet++ models.

| Hyperparameter | Default value |
|---|---|
| Learning rate | 0.0001 |
| Batch size | 128 |
| Size of hidden embeddings | 128 |
| Number of DimeNet++ blocks | 4 |
| Size of initial embedding | 64 |
| Size of basis embedding | 8 |
| Size of output embedding | 256 |
| Number of spherical harmonics basis functions | 7 |
| Number of radial basis functions | 6 |
| Cutoff distance for defining edges | 5.0 Å |
| Maximum number of neighbors (edges per node) | 32 |
| Envelope exponent for smooth cutoff | 5 |
| Number of residual layers before skip connection | 1 |
| Number of residual layers after skip connection | 2 |
| Number of linear layers in DimeNet++ output blocks | 3 |
| Number of linear layers in $MLP_{mol}$, $MLP_{atom}$, $MLP_{bond}$ | 3 |
| Number of linear layers in $MLP_{perm}$ | 2 |
| Activation function | swish |

Training, validation, and test splits and all code associated with the models published herein is available as 3D.zip (https://doi.org/10.6084/m9.figshare.25213742.v3) and on GitHub (https://github.com/nsf-c-cas/AcidAmine_Descriptor_Predict/).

## 5.2 Training Methods

*Surrogate Conformer Generation with ETKDG/MMFF94*

The following workflow was employed to generate the surrogate conformers that were provided as input to the 3D GNNs during training and inference. Given a SMILES, up to 100 conformers were embedded with ETKDG in RDKit[2] using an RMSD distance threshold of 0.25 Å. Each individual conformer then underwent up to 200 steps of MMFF94 optimization in RDKit. The optimized conformer ensembles were then clustered with Butina clustering, using an initial threshold of 0.20 Å. If there were more than 20 conformers remaining after clustering, the ensemble was iteratively re-clustered using increasingly large RMSD thresholds (increments of 0.1 Å) until the total number of clusters was less than or equal to 20. The cluster centroids were then selected to form the final ensembles.

*Training Protocols*
Each DimeNet++ model was trained with a mean squared error (MSE) loss until convergence, or for a maximum of 3000 epochs. The best model over the course of training was selected for downstream evaluation according to the mean absolute error (MAE) on the validation set. We used the Adam optimizer in Pytorch with its default parameters and a constant learning rate.

For molecules that have multiple conformers in their surrogate MMFF94-optimized ensembles, we used the multiple conformers as a form of data augmentation during training, mapping each conformer instance to the same regression target. At the beginning of training, we sampled (without replacement) $N_c$ total conformers from each molecule’s conformer ensemble. If the ensemble had fewer than $N_c$ conformers, then after sampling all possible conformers, we replaced the conformers and repeated sampling until $N_c$ total conformers were obtained. This ensured that flexible molecules with many conformers were not overrepresented during training. After sampling, the same $N_c$ conformers were used for each epoch. For the amines, we used $N_c = 20$. For the acids, we used $N_c = 10$ due to the larger size of the acid datasets.

## 5.3 Analysis of 3D Model Performance on Outliers for the 2D model

**Table S6.** Comparison of 2D vs. 3D GNN descriptor predictions for the outliers highlighted in Figure 3B. Bolded rows indicate outliers for which the 3D model showed markedly improved accuracy.

| Boltzmann-Averaged Descriptor | DFT Descriptor | 2D GNN Prediction | 3D GNN Prediction |
|---|---|---|---|
| Acid NMR $H^5$ | 19.14 | 25.89 | 25.92 |
| Acid NBO $H^5$ | 0.53 | 0.50 | 0.50 |
| **Primary Amine LP Energy $N^1$** | **-0.39** | **-0.42** | **-0.38** |
| Secondary Amine NBO $H^4$ | 0.41 | 0.38 | 0.39 |
| Secondary Amine LP Occupancy $N^1$ | 1.85 | 1.89 | 1.90 |
| **Secondary Amine NMR $H^4$** | **32.34** | **30.48** | **31.40** |

### 5.4 Comparing the Absolute Accuracies ($R^2$) of the 2D and 3D GNNs

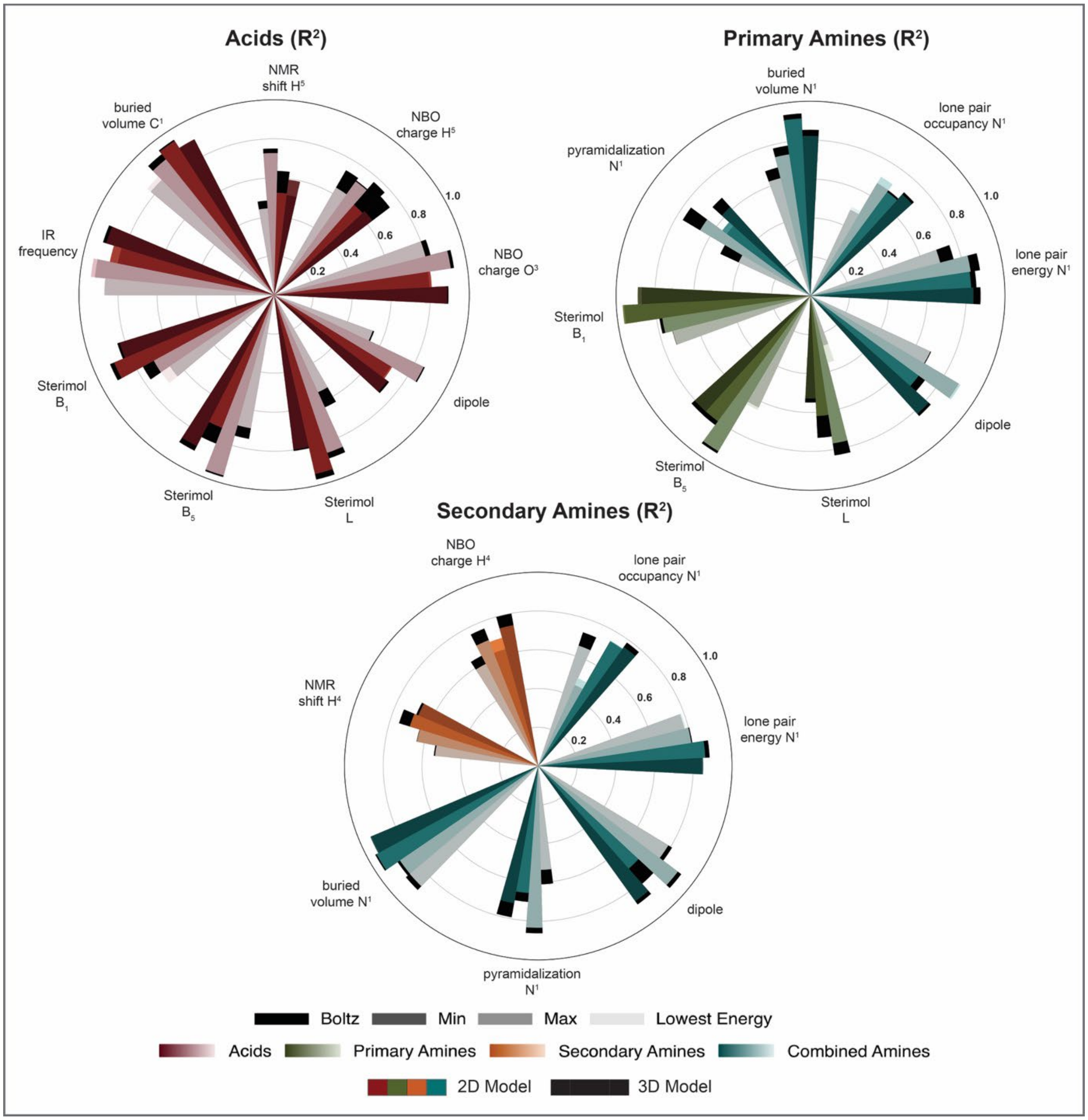


**Figure S15.** Model performance of the 2D GNNs vs. the 3D GNNs on the acids and amines, evaluated as $R^2$ on the test split of each subset. The 2D model results are visualized as colored bars overlaid on the 3D model results, which are visualized as black bars.

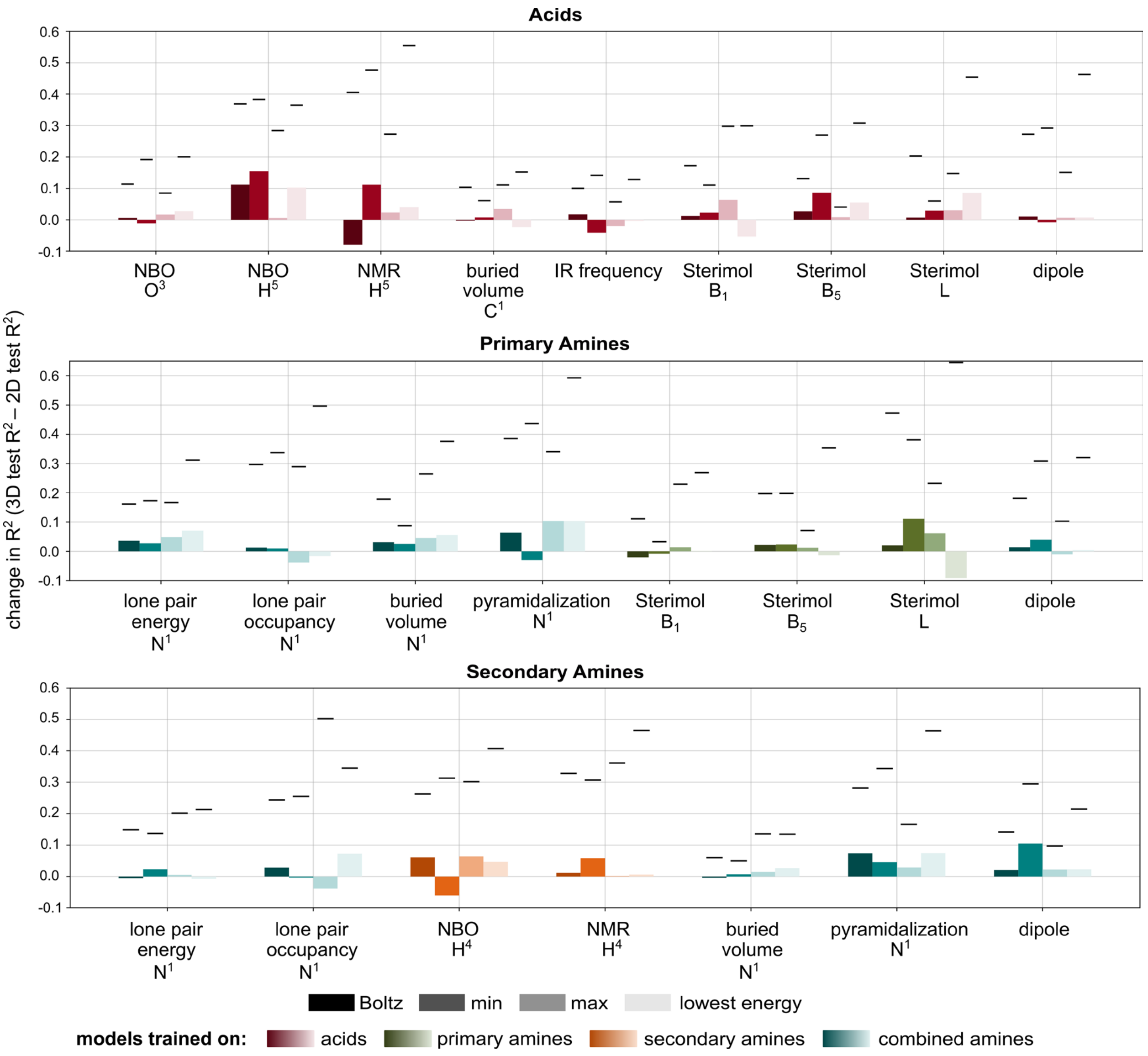


**Figure S16** Reproduced Figure 4 showing improvement in model performance on select molecule-, atom-, and bond-level descriptors when using the 3D GNN vs. the 2D GNN, reported as the change in $R^2$ for the test set. Black lines indicate the maximum achievable improvement 3D GNNs could have compared to 2D GNNs.

A complete comparison of statistics ($R^2$ and MAE) for 2D and 3D model predictions for the test set and the external validation set are included as 2D_and_3D_test_and_ext_val_statistics.xlsx (https://doi.org/10.6084/m9.figshare.25213742.v3).

## 6. Evaluation of Acceptable Error

### 6.1 Models Trained with DFT, 2D, and 3D GNN Predicted

As described in the manuscript, a previously published amide coupling dataset[21] and DFT-level descriptors published herein (DFT-derived or GNN-predicted) were used to evaluate the impact of descriptors on predicted ln(*k*). Of note, the original study employed DFT-level descriptors calculated for the carboxylate anion and the activated carboxylic acid intermediate; for this study, these were replaced with analogous descriptors from the carboxylic acid. **Figure S17A** shows the multivariate linear regression (MVLR) models evaluated with a 70:30 y-equidistant train:test split. In each case, independent of the origin of the descriptors (DFT-derived, 2D GNN predicted, or 3D GNN predicted) used for training, the model statistics are comparable. The same is true for models retrained on the entire dataset for virtual screening (**Figure S17B**).

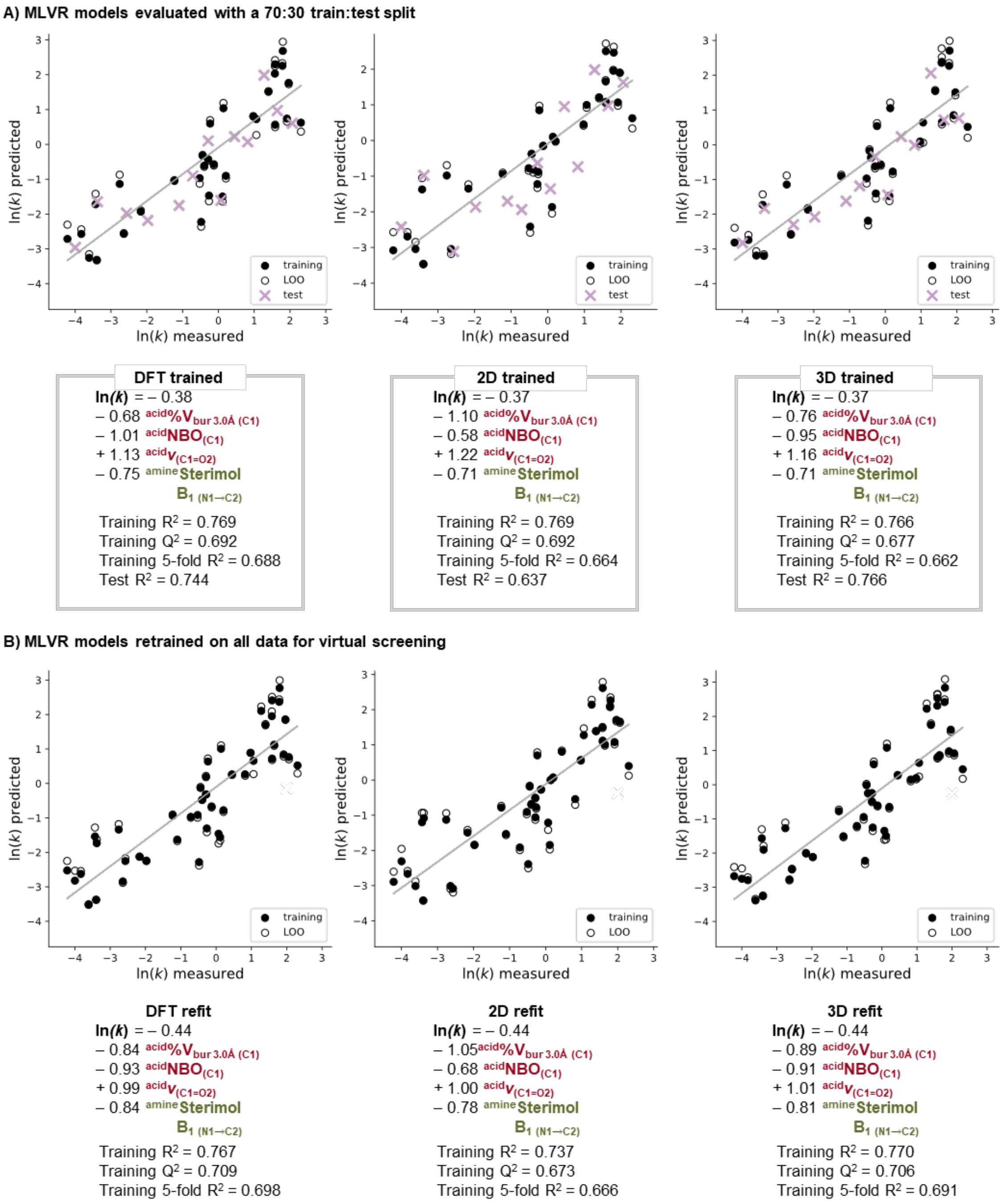


**Figure S17** (A) MVLR amide coupling rate models built with DFT-derived, 2D GNN predicted, and 3D GNN predicted descriptors, evaluated with a 70:30 y-equidistant train:test split. (B) Analogous models refit to all data and used for virtual screening. *Note, the rightmost panel of this figure was presented in the manuscript (Figure 5B) and is reproduced here for direct comparison.

### 6.2 Generating Amide Couplings for Virtual Screening

Amide couplings for virtual screening were generated by pairing carboxylic acids with primary alkyl amines from the test and external validation sets. To ensure that a) every carboxylic acid and primary amine appears in the virtual screening set at least once and that b) the test and external validation set were represented:

- Each carboxylic acid in the test set was paired with a random primary amine from the test set and a random primary amine from the external validation set.
- Each carboxylic acid in the external validation set was paired with a random primary amine from the test set and a random primary amine from the external validation set.
- Each primary amine in the test set was paired with a random carboxylic acid from the test set and a random carboxylic acid from the external validation set.
- Each primary amine in the external validation set was paired with a random carboxylic acid from the test set and a random carboxylic acid from the external validation set.

Duplicate pairings generated from this method were removed. This led to a list of 2362 pairs of acids and amines for virtual screening.

### 6.3 Comparing ln(*k*) Predictions from using DFT, 2D, and 3D GNN Predicted Descriptors

In order to determine if models trained on the same type of descriptors used for virtual screening would account for systematic errors, an extensive series of comparisons was conducted (**Table S7**). We found that the choice of descriptors used to train the model for virtual screening (DFT-derived descriptors or GNN predicted descriptors) had little impact on the integrity of the predictions (**1** vs **3** and **2** vs **4**). In practice, we found no systematic errors were addressed by using 3D-trained models over DFT-trained models when predicting amide coupling rates with 3D descriptors. The same was true for 2D descriptors. We observed modest improvements when 3D GNN predicted descriptors were used compared to when 2D GNN predicted descriptors were used (**1** vs **2** and **3** vs **4**). While $R^2$ are higher for the broader prediction range than for the experimentally feasible range of rates (i.e., $-3.5 < \ln(k) < 2.5$), this is inflated due to the much larger range ($-17.9 < \ln(k) < 7.1$). Improved MAE's are a more accurate representation and indicate a better fit in the experimentally feasible range of rates (i.e.,$-3.5 <$ DFT predicted $\ln(k) < 2.5$).

**Table S7.** Comparisons of statistics for models trained with various descriptor types (i.e., DFT model, 2D model, 3D model) and virtually screened with various descriptor types (i.e., DFT descriptors, 2D descriptors, 3D descriptors).

| **Predictions of ln(*k*) using DFT model & DFT descriptors *compared to* Predictions of ln(*k*) using** | **$R^2$** | **MAE** | **$R^2$** –3.5 < ln(*k*) < 2.5 | **MAE** –3.5 < ln(*k*) < 2.5 |
|---|---|---|---|---|
| **(1)** DFT model & 2D descriptors | 0.836 | 0.729 | 0.699 | 0.536 |
| **(2)** DFT model & 3D descriptors | 0.850 | 0.715 | 0.711 | 0.533 |
| **(3)** 2D model & 2D descriptors | 0.834 | 0.740 | 0.694 | 0.549 |
| **(4)** 3D model & 3D descriptors | 0.849 | 0.719 | 0.692 | 0.573 |

### 6.4 Comparing ln(*k*) Predictions in Different Rate Regimes

From the analysis presented in **Table S7**, it was determined that scenario 4 (predictions of ln(*k*) using DFT model and DFT descriptors compared to predictions of ln(*k*) using the 3D model and 3D descriptors) was the best performing. To further this analysis, we investigated the statistics of predictions in classified rate regimes that are kinetically meaningful (i.e., instant, moderate, and extremely slow)(**Table S8**). We found that the MLR model performs best in the moderate rate regime, where predictions of rate provide greater insight than classifications of slow or fast reactions.

**Table S8.** Comparison of statistics for predictions made in different rate regimes (i.e., instant, moderate, and extremely slow as determined by the DFT predicted ln(*k*)) using analysis presented in **Table S7** (4).

| **Classified Reaction Rate Regime** | **DFT Predicted ln(*k*)** | **$R^2$** | **MAE** |
|---|---|---|---|
| Entire Range | all values | 0.849 | 0.719 |
| Extremely Slow | < –3.5 | 0.585 | 1.369 |
| Moderate | –3.5 < ln(*k*) < 2.5 | 0.692 | 0.573 |
| Instantaneous | < 2.5 | 0.258 | 0.803 |

### 6.5 Acetic Acid as an Outlier

Using 3D GNN predicted descriptors, predictions of ln(*k*) for couplings that included acetic acid were notably different compared to those obtained using DFT-derived or 2D GNN predicted descriptors. Upon investigating the descriptors in the model, we noticed this difference was caused by the poorly predicted 3D GNN Boltzmann averaged IR descriptor (**Table S9**, n=0).

**Table S9.** Comparison of DFT-derived and 3D GNN predicted Boltzmann averaged IR descriptors for a series of alkyl acids.

| 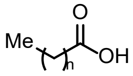 | **n** | **DFT-derived $IR_{Boltz}$** | **2D GNN Predicted $IR_{Boltz}$** | **3D GNN Predicted $IR_{Boltz}$** |
|---|---|---|---|---|
| Acetic | 0 | 1857.5 | 1851.2 | 1979.4 |
| Propionoic | 1 | 1848.0 | 1849.5 | 1844.3 |
| Butyric | 2 | 1845.7 | 1843.7 | 1843.2 |
| Pentanoic | 3 | 1845.3 | 1843.9 | 1844.6 |
| Hexanoic | 4 | 1844.6 | 1844.2 | 1841.2 |
| Heptanoic | 5 | 1843.5 | 1843.8 | 1841.8 |

Figure 5C excludes results with acetic acid due to the 3D GNN significantly poor prediction of the $C^1$-$O^2$ IR frequency (Boltz.) (**Table S9**). Although one might expect the 3D surrogate models to be more accurate on simple structures like acetic acid, acetic acid is an extreme outlier for the 3D GNN due to the few atoms in the molecular graph. Whereas acetic acid has only 4 non-hydrogen atoms, the smallest acid in the training dataset (dibromoacetic acid) has 6 non-hydrogen atoms. This introduces two possible failure modes in the 3D GNN. First, because $\boldsymbol{h}_{agg}$ (**Equation S2**) sums over the learned atom representations, the sum will involve fewer atoms for acetic acid than any example the model is trained on, potentially leading to a distribution shift in the learned representation space. Second, during each message passing layer, there are only 3 edges to $C^1$ in the case of acetic acid, compared to 5 edges in the case of the smallest acid in the training dataset (**Figure S18**). As such, the 3D GNN is never trained to model the scenario where fewer than 5 messages are sent to the $C^1$ atom, again possibly leading to a distribution shift in the learned representations. A similar analysis could be performed for $C^4$, $O^2$, and $O^3$. We note that the 3D GNN is particularly susceptible to this outlier as opposed to the 2D GNN, as edges in the 2D molecular graph only include covalent bonds. Hence, when using the 2D GNN, the $C^1$ atom would have 3 incoming messages in the case of both acetic acid and dibromoacetic acid. Remarkably, when the 3D GNN is applied to predict the IR frequency for alkyl acids with longer alkyl chains (propionic acid, butyric acid, etc.), the 3D GNN's predictions of the IR frequency (Boltz.) change dramatically (**Table S9**, n=1–5). The predictions for the larger alkyl acids also match the DFT-computed IR frequency (Boltz.) for acetic acid much more closely, empirically demonstrating that acetic acid is a unique failure mode for the 3D GNN given our training set.

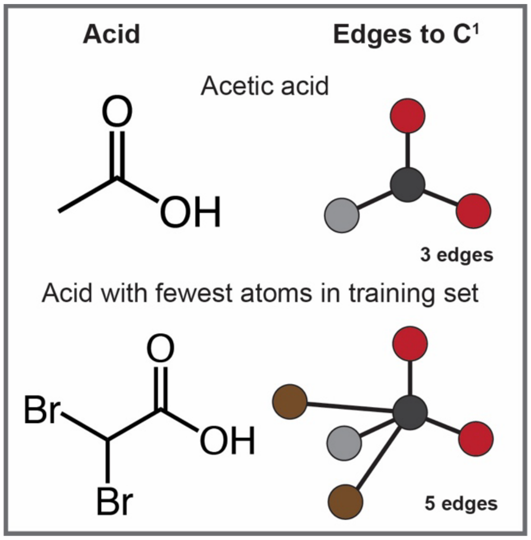


**Figure S18.** Comparison of the molecular graphs for acetic acid vs. dibromoacetic acid, the acid in the training set containing the fewest number of atoms. Whereas acetic acid has three 3D edges to $C^1$, dibromoacetic acid five such edges, possibly introducing a distribution shift that severely worsens model performance specifically on acetic acid.

## 6. SI References


1. Corsello, S. M.; Bittker, J. A.; Liu, Z.; Gould, J.; McCarren, P.; Hirschman, J. E.; Johnston, S. E.; Vrcic, A.; Wong, B.; Khan, M.; Asiedu, J.; Narayan, R.; Mader, C. C.; Subramanian, A.; Golub, T. R. The Drug Repurposing Hub: a next-generation drug library and information resource. *Nat. Med.* **2017**, *23*, 405–408.
2. RDKit: Open-Source Cheminformatics Software. https://www.rdkit.org/
3. Schrödinger Release 2021-4: MacroModel, Schrödinger, LLC, New York, NY, **2021**.
4. Lu, C.; Wu, C.; Ghoreishi, D.; Chen, W.; Wang, L.; Damm, W.; Ross, G. A.; Dahlgren, M. K.; Russell, E.; Von Bargen, C. D.; et al. OPLS4: Improving Force Field Accuracy on Challenging Regimes of Chemical Space. *J. Chem. Theory Comput.* **2021**, *17*, 4291–4300.
5. Kelley, L. A.; Gardner, S. P.; Sutcliffe, M. J. An automated approach for clustering an ensemble of NMR-derived protein structures into conformationally related subfamilies. *Protein Eng. Des. Sel.* **1996**, *9*, 1063–1065.
6. Gaussian 16, Revision C.01. Frisch, M. J., Trucks, G. W., Schlegel, H. B., Scuseria, G. E., Robb, M. A., Cheeseman, J. R., Scalmani, G., Barone, V., Petersson, G. A., Nakatsuji, H., Li, X., Caricato, M., Marenich, A. V., Bloino, J., Janesko, B. G., Gomperts, R., Mennucci, B., Hratchian, H. P., Ortiz, J. V., Izmaylov, A. F., Sonnenberg, J. L., Williams-Young, D., Ding, F., Lipparini, F., Egidi, F., Goings, J., Peng, B., Petrone, A., Henderson, T., Ranasinghe, D., Zakrzewski, V. G., Gao, J., Rega, N., Zheng, G., Liang, W., Hada, M., Ehara, M., Toyota, K., Fukuda, R., Hasegawa, J., Ishida, M., Nakajima, T., Honda, Y., Kitao, O., Nakai, H., Vreven, T., Throssell, K., Montgomery, J. A. J., Peralta, J. E., Ogliaro, F., Bearpark, M. J., Heyd, J. J., Brothers, E. N., Kudin, K. N., Staroverov, V. N., Keith, T. A., Kobayashi, R., Normand, J., Raghavachari, K., Rendell, A. P., Burant, J. C., Iyengar, S. S., Tomasi, J., Cossi, M., Millam, J. M., Klene, M., Adamo, C., Cammi, R., Ochterski, J. W., Martin, R. L., Morokuma, K., Farkas, O., Foresman, J. B., Fox, D. J.; Gaussian, Inc., Wallingford, CT, **2016**.
7. Tirado-Rives, J.; Jorgensen, W. L. Performance of B3LYP Density Functional Methods for a Large Set of Organic Molecules..*J. Chem. Theory Comput.* **2008**, *4*, 297–306.
8. Grimme, S.; Ehrlich, S.; Goerigk, L. Effect of the damping function in dispersion corrected density functional theory. *J. Comput. Chem.* **2011**, *32*, 1456–1465.
9. Ditchfield, R.; Hehre, W. J.; Pople, J. A. Self-Consistent Molecular-Orbital Methods. IX. An Extended Gaussian-Type Basis for Molecular-Orbital Studies of Organic Molecules. *J. Chem. Phys.* **1971**, *54*, 724–728.
10. Wadt, W. R.; Hay, P. J. *Ab initio* effective core potentials for molecular calculations. Potentials for main group elements Na to Bi. *J. Chem. Phys.* **1985**, *82*, 284–298.
11. Zhao, Y.; Truhlar, D. G. The M06 suite of density functionals for main group thermochemistry, thermochemical kinetics, noncovalent interactions, excited states, and transition elements: two new

functionals and systematic testing of four M06-class functionals and 12 other functionals. *Theor. Chem. Acc.* **2008**, *120*, 215–224.

12. Weigend, F.; Ahlrichs, R. Balanced basis sets of split valence, triple zeta valence and quadruple zeta valence quality for H to Rn: Design and assessment of accuracy. *Phys. Chem. Chem. Phys.* **2005**, *7*, 3297–3305.
13. Dunning, T. H. Jr.; Hay, P. J. In *Modern Theoretical Chemistry*, Vol. 3; Schaefer, H. F. III, Ed.; Plenum, 1977. pp 1–28.
14. Glendening, E. D.; Badenhoop, J. K.; Reed, A. E.; Carpenter, J. E.; Bohmann, J. A.; Morales, C. M.; Karafiloglou, P.; Landis, C. R.; Weinhold, F. NBO 7.0.
15. Ditchfield, R. Self-consistent perturbation theory of diamagnetism, *Mol. Phys*. **1974**, *27*, 789–807.
16. Haas, B. C., Hardy, M. A. SigmanGroup/Get_Properties: Get_Properties_v1.0.3 (v1.0.3). *Zenodo* **2024**. DOI: 10.5281/zenodo.10651727
17. Dobson, C. M. Chemical space and biology. *Nature* **2004**, *432*, 824–828.
18. McInnes, L.; Healy, J.; Melville, J. UMAP: Uniform Manifold Approximation and Projection for Dimension Reduction, v3. *arXiv* **2020**. DOI: https://arxiv.org/abs/1802.03426v3.
19. Gasteiger, J.; Giri, S.; Margraf, J. T.; Günnemann, S. Fast and Uncertainty-Aware Directional Message Passing for Non-Equilibrium Molecules, v.3. *arXiv* **2020**. DOI: https://arxiv.org/abs/2011.14115v3.
20. Gasteiger, J.; Janek Groß, J.; Günnemann, S. Directional Message Passing for Molecular Graphs, v.1. *arXiv* **2020**. DOI: https://arxiv.org/abs/2003.03123v2.
21. Haas, B. C.; Goetz, A. G.; Bahamonde, A.; McWilliams, J. C.; Sigman, M. S. Predicting relative efficiency of amide bond formation using multivariate linear regression. *Proc. Natl. Acad. Sci. U. S. A.* **2022**, *119*, e2118451119.
